# Supplementary material for: Intestinal barrier dysfunction plays an integral role in arthritis pathology and can be targeted to ameliorate disease
Source: Med. 2021 Jul 9;2(7):864–883.e9. doi: 10.1016/j.medj.2021.04.013 (PMC8280953; doi:10.1016/j.medj.2021.04.013)
Supplement: Document S1. Table S1 and Figures S1–S6 [file mmc1.pdf]

Med, Volume *2*

Supplemental information

Intestinal barrier dysfunction plays an integral role in arthritis pathology and can be targeted to ameliorate disease

Diana E. Matei, Madhvi Menon, Dagmar G. Alber, Andrew M. Smith, Bahman Nedjat-Shokouhi, Alessio Fasano, Laura Magill, Amanda Duhlin, Samuel Bitoun, Aude Gleizes, Salima Hacein-Bey-Abina, Jessica J. Manson, Elizabeth C. Rosser, The ABIRISK Consortium, Nigel Klein, Paul A. Blair, and Claudia Mauri

Supplemental Table 1. Summary data of patients and healthy controls. Related to Figure 1.

**Index**	**RA (n=167)**	**Early RA (n=8)**	**Pre-RA (n=9)**	**HC (n=34)**
Female (%)	77.77%	100%	100%	70.83%
Age	52.97±15.88	44.29±8.95	51.143±14.4	49.94±17.6
Smoking	21.66% (n=60)	Unk	Unk	27.27% (n=22)
Infections	3 (2.38%)	0	0	N/A
Metabolic disorders	11 (8.73%)	3 (42.86%)	2 (28.57%)	N/A
ESR (mm/h)	17.22±20.42	25.5±7.5 (6 Unk)	N/A	N/A
CRP (g/L)	7.44±15.91	12.54±19.12	N/A	N/A
DAS28 score	3.46±1.62	2.725±0.275 (6 Unk)	N/A	N/A
RF+	80.36% (n=56)	87.5%	88.89%	N/A
CCP+	73.21% (n=56)	100%	44.45%	N/A
**Treatments**				
NSAIDs	18 (10.78%)	1 (12.5%)	1 (11.11%)	N/A
DMARDs	101 (60.47%)	2 (25%)	2 (22.22%)	N/A
Glucocorticoids	19 (11.38%)	1 (12.5%)	0	N/A
Biologics - Anti-TNFα (Adalimumab, Infliximab, Etanercept)	46 (27.55%)	0	0	N/A
Biologics - Anti-IL-6R (Tociluzimab)	7 (4.19%)	0	0	N/A
Biologics - Anti-CD20 (Rituximab)	7 (4.19%)	0	0	N/A
Untreated	18 (10.78%)	4 (50%)	6 (66.67%)	N/A

Unk- Unknown, ESR- erythrocyte sedimentation rate, CRP- C-reactive protein, DAS28- 28 Joint disease activity score, RF- rheumatoid factor, CCP - cyclic citrullinated peptide, NSAID- non-steroidal anti-inflammatory drug, DMARD- disease modifying anti-rheumatic drugs.

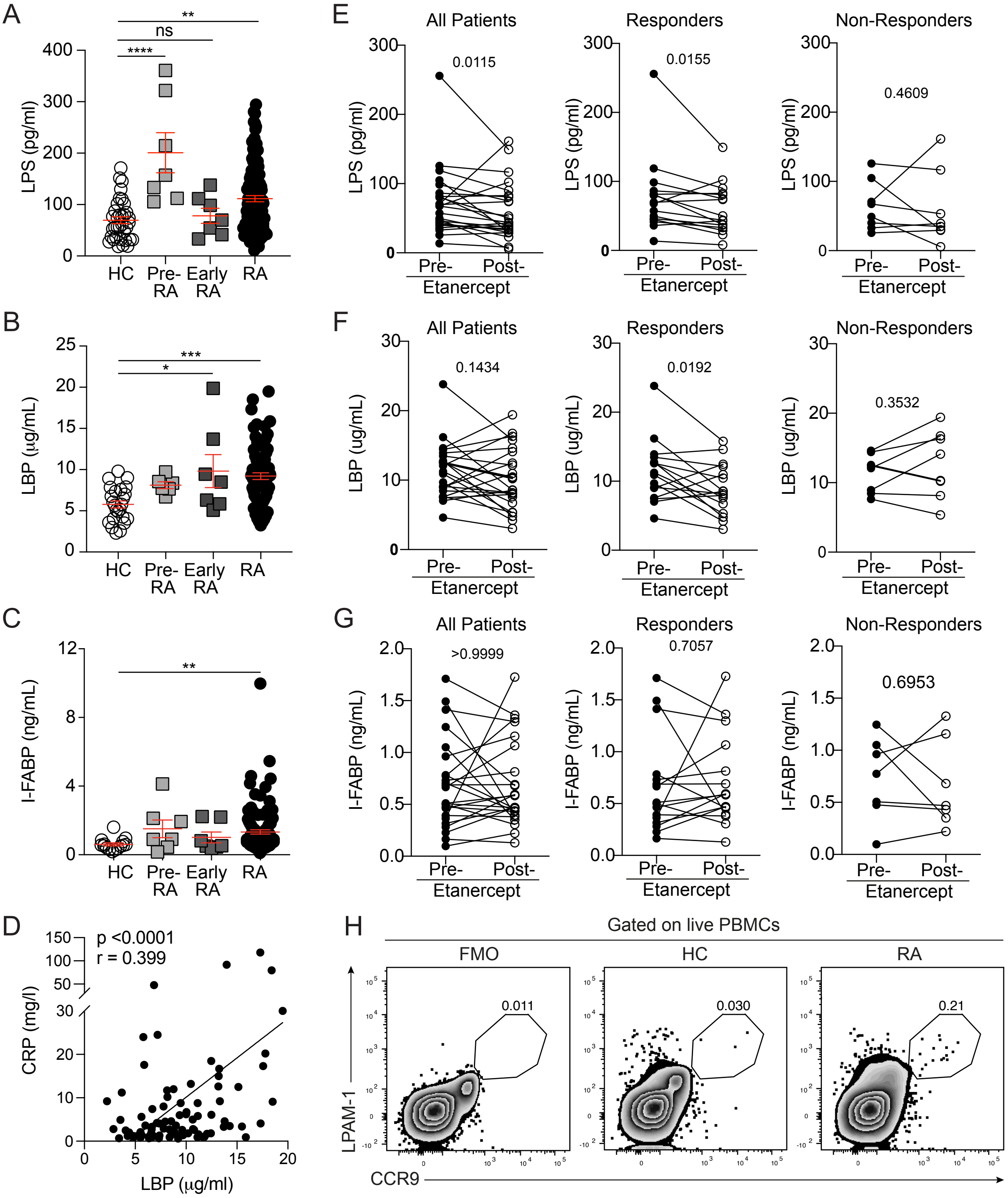

Supplemental Figure 1

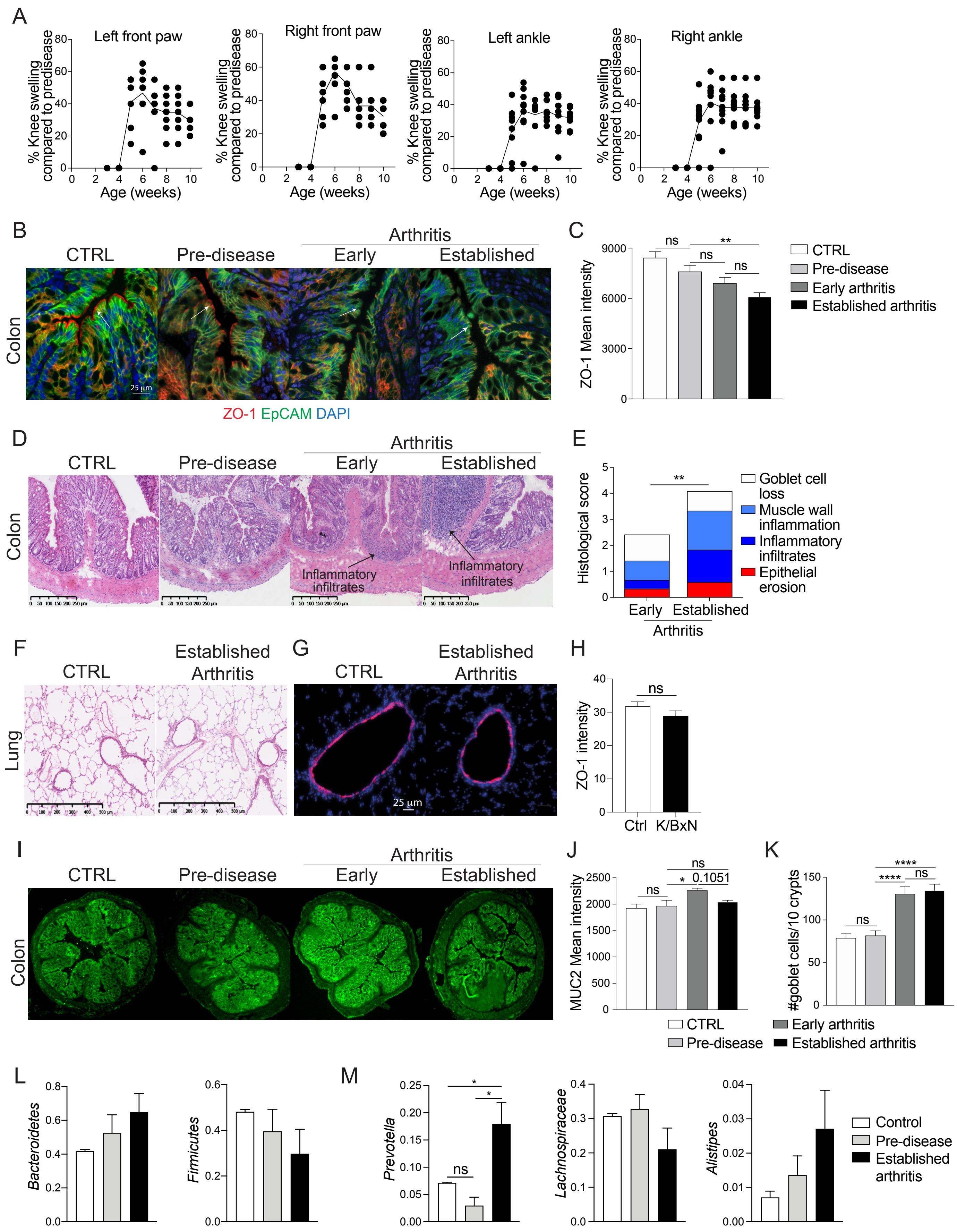

Supplemental Figure 2

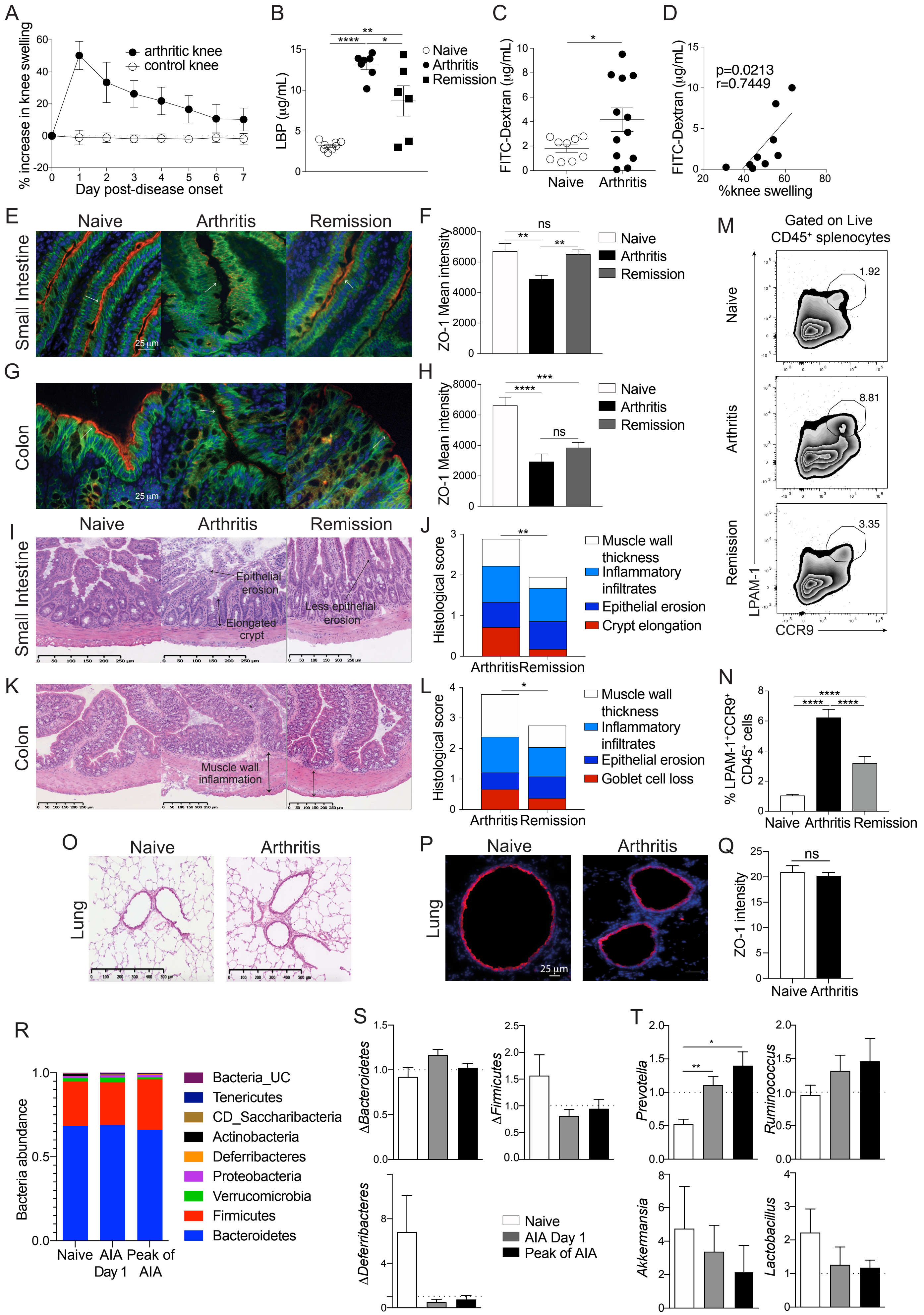

Supplemental Figure 3

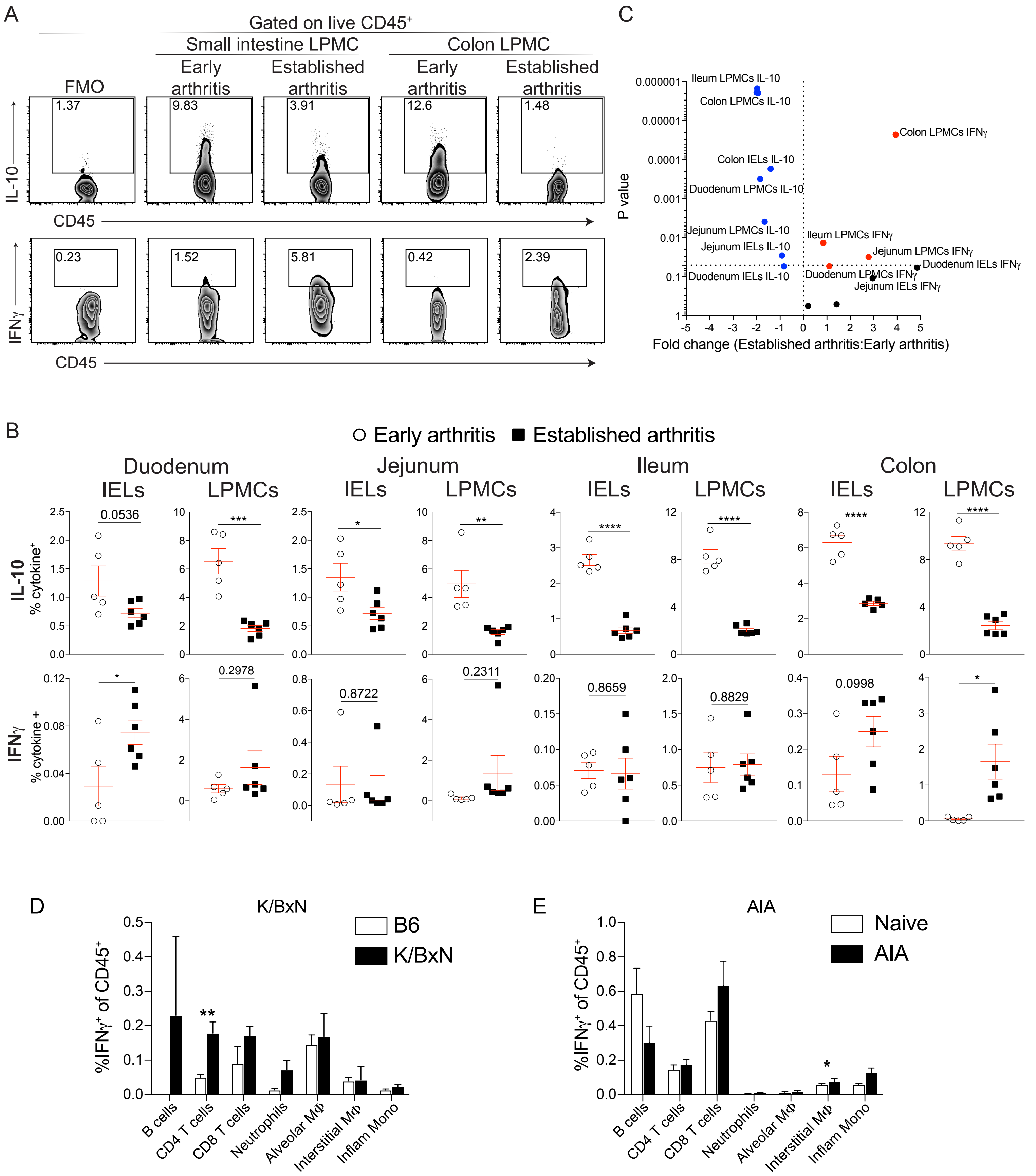

Supplemental Figure 4

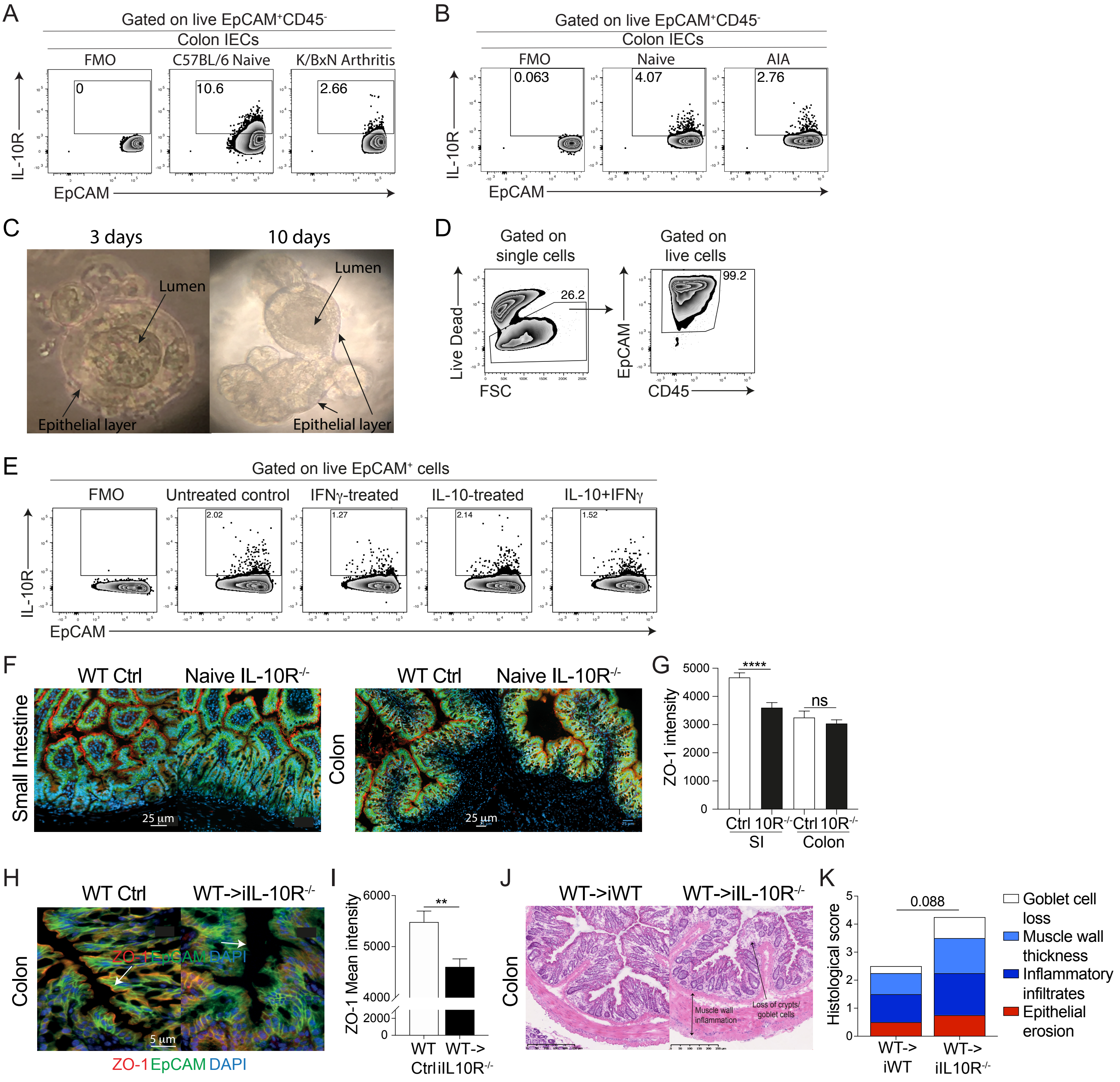

Supplemental Figure 5

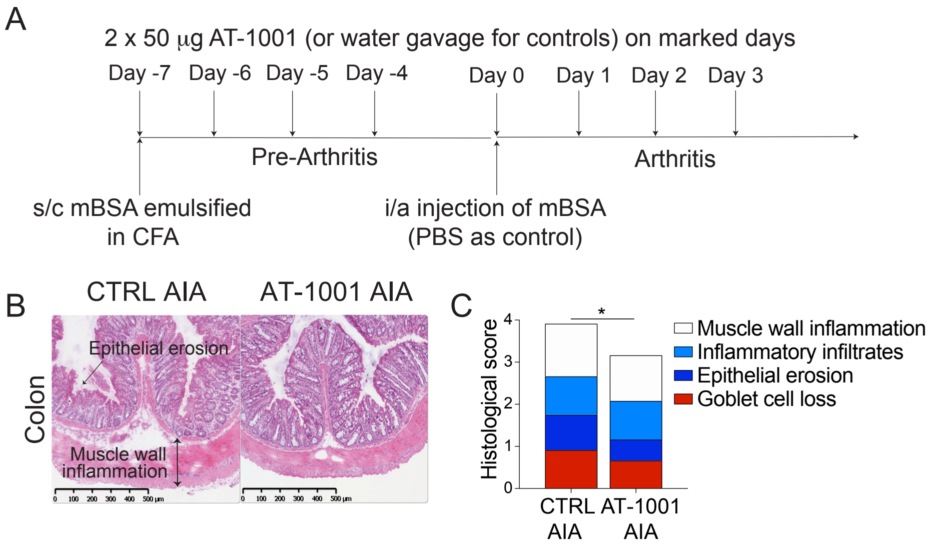

Supplemental Figure 6

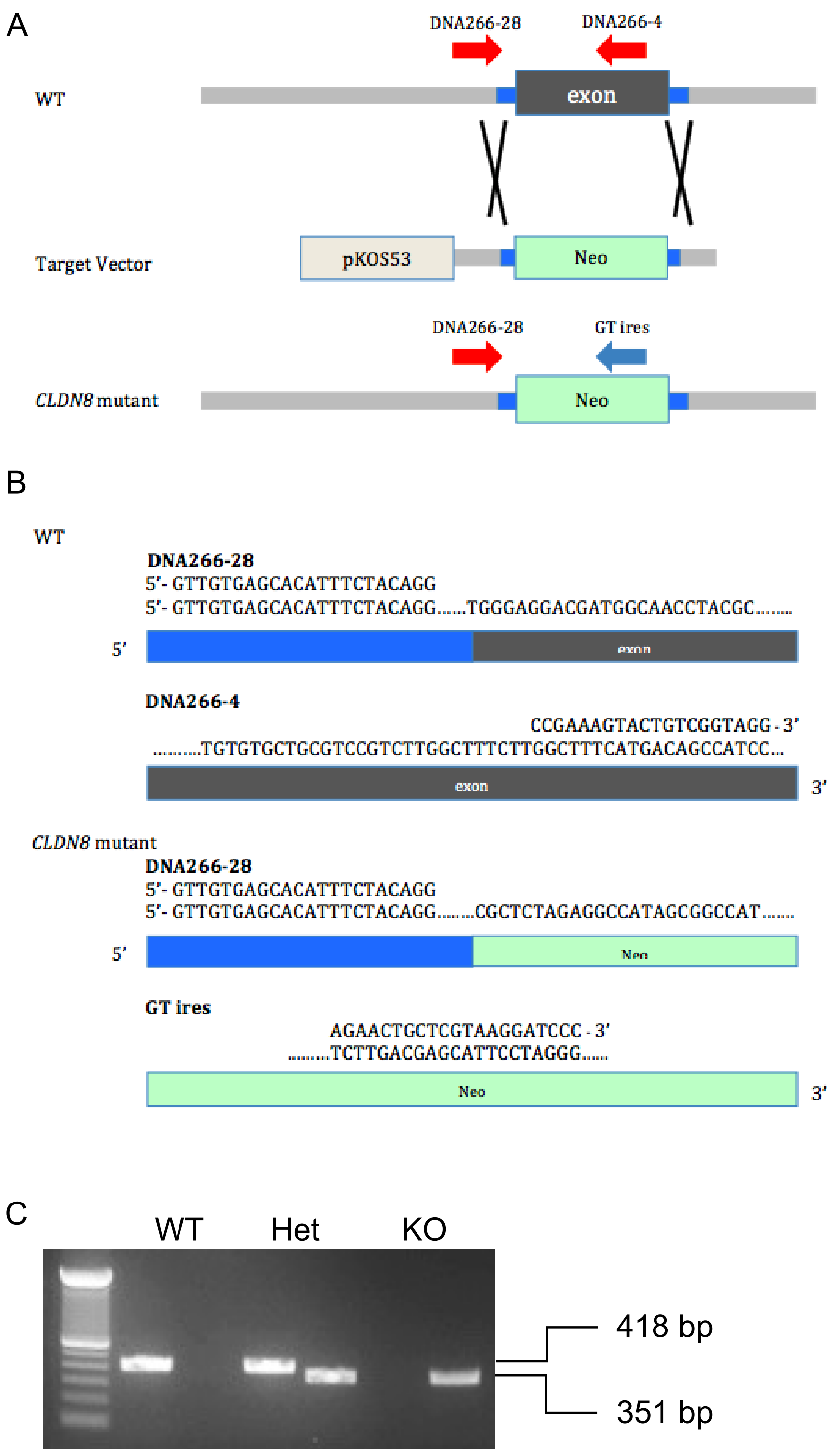

Supplemental Methods Figure 1

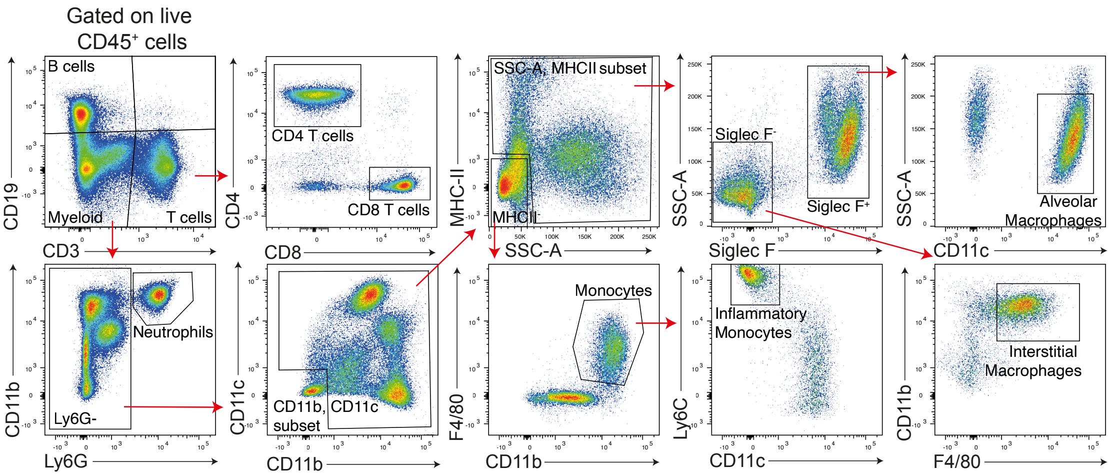

Supplemental Methods Figure 2

Supplemental Figure 1. NSAIDs do not have a significant effect on intestinal permeability, whereas etanercept partly corrects it. Related to Figure 1. (A-C) Concentration of **(A)** LPS, **(B)** LPS-binding protein (LBP) and **(C)** I-FABP in the serum of healthy individuals (HC, n=34) and in patients with pre-rheumatoid arthritis (pre-RA; n=7), early RA (n= 7) and RA patients excluding those treated with NSAIDs (n=99), as measured by ELISA. **(D)** Correlation between LBP and CRP levels in the serum of RA patients (n=124). **(E-G)** Concentration of (E)LPS, **(F)** LBP, and **(G)** I-FABP in the serum of RA patients pre- and post-etanercept treatment (approximately 6 months post-treatment). Left – all patients (n=24), Centre – treatment responders (ΔDAS28>1.2, n=16), Right – non-responders (ΔDAS28<1.2, n=8). **(H)** Representative FACS plots displaying LPAM-1^{+}CCR9^{+} live peripheral blood mononuclear cells (PBMC) isolated from RA patients and healthy controls (HC). *P < 0.05, **P < 0.01, ***P < 0.001. (**A, B, C**) one-way ANOVA with multiple comparisons calculated by Tukey's test. (**D**) Pearson's correlation. (**E, F, G**) paired t test. Data represent mean ± S.E.M.

Supplemental Figure 2. Mice with arthritis display colonic inflammation and gut microbial dysbiosis. Related to Figure 2 and Figure 3. (A) Representative time course of knee swelling in K/BxN mice with spontaneous arthritis. Knee swelling displayed as a percentage increase of knee size as compared to pre-disease initiation (n=5/group). **(B-E) (B)** Representative ZO-1 staining, **(C)** mean ZO-1intensity, **(D)** representative H&E staining and **(E)** histological scores for the colon of K/BxN mice throughout arthritis development and compared to naïve C57BL/6 control. **(F-H) (F)** Representative H&E staining, **(G)** representativeZO-1 staining, and **(H)** mean ZO-1 intensity for lungs from K/BxN mice with established arthritis and CTRL naïve mice. **(I-K) (I)** Representative MUC2 staining, **(J)** mean MUC2 intensity and **(K)** goblet cell counts from PAS staining for the colon of K/BxN mice throughout arthritis development and compared to naïve C57BL/6 control. **(L)** Bar charts showing the abundance of the noted bacterial phyla in samples collected from K/BxN mice at pre-disease (3 wk old, n=4) and established arthritis (6 wk old, n=4) stages, compared to Control (KRN, n=3). **(M)** Bar charts showing the abundance of the noted bacterial genera in samples collected from K/BxN mice at pre-disease (3 wk old, n=4) and established arthritis (6 wk old, n=4) stages, compared to Control (KRN, n=3). *P < 0.05, **P< 0.01, ****P < 0.0001, ns: no significance. (**C, J, K, L, M**) one-way ANOVA, (**E**) two-way ANOVA, (**H**) unpaired t test. For panels **A-K** one of two experiments is shown. Data represent mean ± S.E.M.

Supplemental Figure 3. Mice with AIA display increased gut permeability and inflammation and gut microbial dysbiosis from day 1 of arthritis. Related to Figure 2 and Figure 3. (A) Representative time course of knee swelling in AIA in C57BL/6 WT mice. Knee swelling displayed as percentage increase of knee size as compared to pre-disease induction (n=5/group). **(B)** Concentration of LBP in the serum of WT naïve, arthritic mice (AIA day 3), and mice in remission from arthritis (AIA day 7) (n=5/group). **(C)** Levels of FITC-dextran in the serum of WT naïve (n=9) and arthritic (n=12) mice (AIA day 3). **(D)** Correlation between FITC-dextran in the serum and %knee swelling in mice at day 3 of AIA (n=9). **(E-L) (E, G)** Representative ZO-1 staining, **(F, H)** mean ZO-1 intensity, **(I, K)** representative H&E staining and **(J, L)** histological scores for SI and colon, respectively, from WT naïve and arthritic mice

(AIA day 3) and mice in remission from arthritis (AIA day 7). **(M, N)** **(M)** Representative FACS plots and **(N)** bar chart showing the mean expression of LPAM-1 and CCR9 by total CD45$^+$ splenocytes from naïve WT mice, arthritic mice, and mice in remission from arthritis (n=3/group). **(O-Q)** **(O)** Representative H&E staining, **(P)** representative ZO-1 staining, and **(Q)** mean ZO-1 intensity for lungs from WT naive and arthritic mice (AIA day 3). **(R)** Bar chart showing relative bacterial composition at phylum level in samples collected from mice at AIA day 1 and Peak of AIA (day 3), compared to naive mice. **(S)** Bar charts showing the change in the abundance of the noted bacterial phyla between samples collected at AIA day 1 from naive and arthritic mice, and at Peak of AIA from arthritic mice, compared to their abundance in the same mice at days 1-4 (prior to arthritis induction). **(T)** Bar charts showing the abundance of the noted bacterial genera in samples collected at AIA day 1 and Peak of AIA (day 3), compared to naive mice. (n=10/group). *P < 0.05, **P < 0.01, ***P < 0.001, ****P < 0.0001, ns: no significance. (**B, F, H, N, S, T**) one-way ANOVA, (**C, Q**) unpaired t test, (**D**) Pearson's correlations, (**J, L**) two-way ANOVA. UC - Unclassified, CD - Candidatus. For panels **A-Q** one of two experiments is shown. Data represent mean ± S.E.M.

Supplemental Figure 4. Increased IFNγ and decreased IL-10 expression in the guts of arthritic K/BxN mice. Related to Figure 4. (A) Representative FACS plots displaying cytokine production by total CD45$^+$ lamina propria mononuclear cells (LPMC) isolated from the small intestine or colon of early-disease K/BxN mice and K/BxN mice with established arthritis. **(B)** Graphs displaying cytokine expression and **(C)** volcano plot showing the fold change in cytokine expression by total CD45$^+$ intra-epithelial lymphocytes (IEL) and lamina propria mononuclear cells (LPMC) isolated from the noted tissues of early-disease K/BxN mice (n=5) and mice with established arthritis (n=6), as assessed by flow cytometry. **(D, E)** Bar charts showing IFNγ expression by the noted immune cell subsets isolated from lungs of **(D)** K/BxN and C57BL/6 control mice, and from **(E)** C57BL/6 arthritic and naive mice (n=5/group), as assessed by flow cytometry. MΦ- Macrophage; Inflam Mono- Inflammatory Monocytes.*P < 0.05, **P < 0.01, ***P < 0.001, ****P < 0.0001 (**B**, **D**, **E**, unpaired t test). Data represent mean ± S.E.M.

Supplemental Figure 5. IL-10R expression by IECs ex vivo and organoids in vitro, ZO-1 expression on global IL-10R-/- and gut inflammation in non-hematopoietic cell IL-10R-/- chimeric mice. Related to Figure 4. (A) Representative FACS plots of IL-10R on colonic intestinal epithelial cells in naïve C57BL/6 control versus K/BxN arthritic mice and in **(B)** naïve versus arthritic (day 3 of AIA) C57BL/6 mice. **(C)** Representative brightfield scans of growing organoids: left - budding organoids after 3 days in culture, right - mature organoids after approximately 10 days in culture. **(D)** Representative FACS plots showing organoid survival (left) and epithelial cell purity (right). **(E)** Representative FACS plots showing IL-10R expression by epithelial cells isolated from organoids treated with recombinant IFNγ and/or IL-10, or left untreated. **(F, G)** Representative ZO-1 staining and mean ZO-1 intensity from SI and colon of naïve IL-10R-/- mice and WT control. **(H, I)** Representative ZO-1 staining and mean ZO-1 intensity from colon of naïve non-hematopoietic cell IL-10R$^{-/-}$ chimeric mice (WT->iIL-10R$^{-/-}$) and WT control. **(J, K)** Representative H&E staining and histological scores of the colon of WT->iIL-10R$^{-/-}$ and WT->iWT chimeric mice on day 3 of arthritis. **P < 0.01, ****P < 0.0001. (**G, I**) unpaired t test, (**K**) two-way ANOVA. For all panels one of two experiments is shown. Data represent mean ± S.E.M.

Supplemental Figure 6. Modulating gut permeability using AT-1001 improves gut pathology. Related to Figure 6. (A) Schematic of AT-1001 treatment regime for mice during induction of arthritis (s/c- subcutaneous, i/a- intra-articular). **(B)** Representative H&E staining and **(C)** mean histological scores of the colon of untreated and AT-1001-treated arthritic mice (AIA day 3). *P < 0.05. (**C**) two-way ANOVA. For all panels one of two experiments is shown. Data represent mean ± S.E.M.

Supplemental Methods Figure 1. Generation of Cldn8$^{-/-}$ mouse. Related to STAR methods Mice section. (A) Schematic of the targeting strategy for Cldn8$^{-/-}$ mice. WT Cldn8 gene has 1 exon. The target vector, pKOS-53 with a LacZ/Neo cassette, was targeted to Cldn8 gene by homologous recombination. **(B)** Primers DNA266-28, DNA266-4, and GT ires were used to distinguish WT alleles (418 bps) from mutant alleles (351 bps). **(C)** PCR verification of mouse Cldn8 gene deletion from an ear punch biopsy using primers DNA266-28, DNA266-4, and GT ires.

Supplemental Methods Figure 2. Gating strategy for immune cells isolated from arthritic and control lungs. Related to STAR methods Cell Preparation section. Representative FACS plots of subset gating for lymphocytes isolated from the lung of a naive C57BL/6 mouse.
