# Supplementary material for: Intestinal barrier dysfunction plays an integral role in arthritis pathology and can be targeted to ameliorate disease
Source: Med. 2021 Jul 9;2(7):864–883.e9. doi: 10.1016/j.medj.2021.04.013 (PMC8280953; doi:10.1016/j.medj.2021.04.013)
Supplement: Document S2. Article plus supplemental information [file mmc2.pdf]

Med

CellPress OPEN ACCESS

Clinical and Translational Article

Intestinal barrier dysfunction plays an integral role in arthritis pathology and can be targeted to ameliorate disease

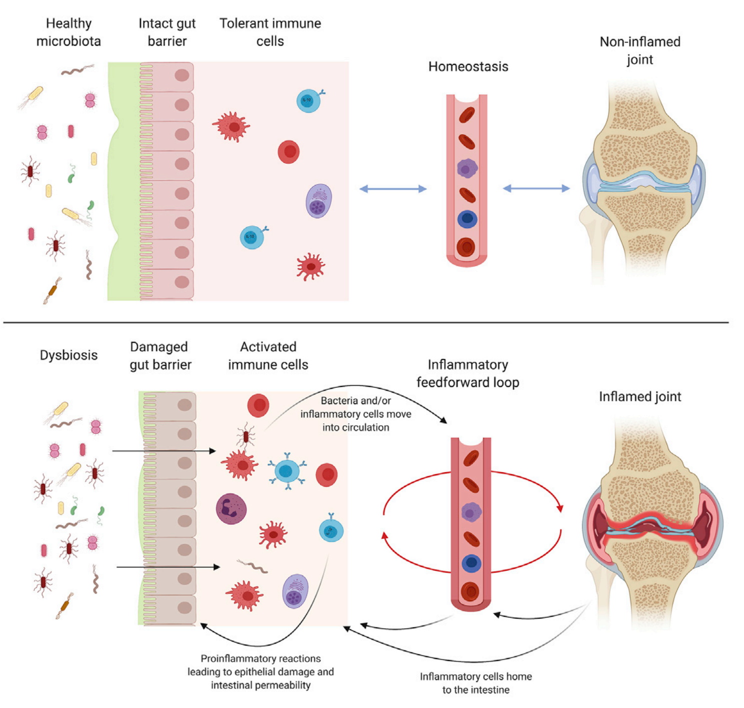

Changes to the gut bacteria have been associated with the development of arthritis; however, the mechanistic connection with disease remains unknown. Matei et al. identify pathological changes to the gut tissue in arthritis, including loss of gut-barrier integrity and inflammatory-cell infiltration, and show that restoration of gut homeostasis ameliorates disease.

Pre-clinical Research

Diana E. Matei, Madhvi Menon, Dagmar G. Alber, ..., Nigel Klein, Paul A. Blair, Claudia Mauri

p.blair@ucl.ac.uk (P.A.B.)
c.mauri@ucl.ac.uk (C.M.)

Highlights

Serum gut-permeability markers LPB, LPS, and I-FABP are increased in RA

Mice with arthritis have increased gut permeability and intestinal inflammation

Both bacteria and leukocytes are needed to disrupt gut-barrier integrity

Prevention of gut-barrier dysfunction in arthritis ameliorates joint inflammation

Matei et al., Med *2*, 864–883
July 9, 2021 © 2021 The Author(s). Published by Elsevier Inc.
https://doi.org/10.1016/j.medj.2021.04.013

CellPress OPEN ACCESS

Med

Clinical and Translational Article

Intestinal barrier dysfunction plays an integral role in arthritis pathology and can be targeted to ameliorate disease

Diana E. Matei,[1] Madhvi Menon,[1,2,3,4] Dagmar G. Alber,[5] Andrew M. Smith,[6] Bahman Nedjat-Shokouhi,[6,7] Alessio Fasano,[8] Laura Magill,[1] Amanda Duhlin,[1] Samuel Bitoun,[9] Aude Gleizes,[10,11] Salima Hacein-Bey-Abina,[10,12] Jessica J. Manson,[13] Elizabeth C. Rosser,[1,14] The ABIRISK Consortium, Nigel Klein,[5] Paul A. Blair,[1,15,16,*] and Claudia Mauri[1,15,*]

SUMMARY

Background: Evidence suggests an important role for gut-microbiota dysbiosis in the development of rheumatoid arthritis (RA). The link between changes in gut bacteria and the development of joint inflammation is missing. Here, we address whether there are changes to the gut environment and how they contribute to arthritis pathogenesis.

Methods: We analyzed changes in markers of gut permeability, damage, and inflammation in peripheral blood and serum of RA patients. Serum, intestines, and lymphoid organs isolated from K/BxN mice with spontaneous arthritis or from wild-type, genetically modified interleukin (IL)-10R$^{-/-}$ or claudin-8$^{-/-}$ mice with induced arthritis were analyzed by immunofluorescence/histology, ELISA, and flow cytometry.

Findings: RA patients display increased levels of serum markers of gut permeability and damage and cellular gut-homing markers, both parameters positively correlating with disease severity. Arthritic mice display increased gut permeability from early stages of disease, as well as bacterial translocation, inflammatory gut damage, increases in interferon γ (IFNγ)$^{+}$ and decreases in IL-10^{+} intestinal-infiltrating leukocyte frequency, and reduced intestinal epithelial IL-10R expression. Mechanistically, both arthritogenic bacteria and leukocytes are required to disrupt gut-barrier integrity. We show that exposing intestinal organoids to IFNγ reduces IL-10R expression by epithelial cells and that mice lacking epithelial IL-10R display increased intestinal permeability and exacerbated arthritis. Claudin-8$^{-/-}$ mice with constitutively increased gut permeability also develop worse joint disease. Treatment of mice with AT-1001, a molecule that prevents development of gut permeability, ameliorates arthritis.

Conclusions: We suggest that breakdown of gut-barrier integrity contributes to arthritis development and propose restoration of gut-barrier homeostasis as a new therapeutic approach for RA.

Funding: Funded by Versus Arthritis (21140 and 21257) and UKRI/MRC (MR/T000910/1).

Context and significance

Rheumatoid arthritis is an autoimmune disorder characterized by chronic joint inflammation. Accumulating evidence suggests that changes in the composition of the bacteria residing in the gut could be responsible for joint inflammation. Currently, it is unclear how bacteria or their products instruct cells of the immune system to become harmful and induce arthritis. Researchers at University College London have shown that, in arthritis, there is profound damage to the gut lining, which fails to work properly as a barrier, as well as an accumulation in the gut of white blood cells that cause inflammation. The authors show that, in arthritis, bacteria cross the prohibited border of the intestinal lining and that repairing gut permeability defects with specific drugs inhibits joint inflammation.

INTRODUCTION

Rheumatoid arthritis (RA) is a systemic chronic autoimmune disorder characterized by a persistent inflammation that results in an aggressive synovitis and destruction

 © 2021 The Author(s). Published by Elsevier Inc.
This is an open access article under the CC BY license (http://creativecommons.org/licenses/by/4.0/).

CellPress
OPEN ACCESS

of joint cartilage and bone.[1] Despite our improved understanding of the genetic and environmental factors that contribute to RA, the etiopathology of disease is still not well understood.[1] There has been a considerable interest in gut microbiota dysbiosis as a possible driver of disease in RA.[2–4] Abnormal expansion of *Prevotella copri*, for instance, has been associated with the pathogenesis of RA.[4–7] The abundance of *P. copri* has been reported to be raised in the feces of treatment-naive new-onset RA patients,[4] *P. copri*-derived peptides have been shown to increase Th1 responses in the peripheral blood mononuclear cells (PBMCs) of these patients,[8] and transfer of *P. copri* to germ-free mice has been suggested to be sufficient to induce joint swelling in the SKG model of spondyloarthritis.[6] Dysbiosis in RA has been hypothesized to contribute to disease pathogenesis by a number of mechanisms, including providing a source of citrullinated peptides,[9] antigenic mimicry,[10] activation of antigen-presenting cells and T cells,[11] and potentially by altering the integrity of the intestinal epithelium.[12]

The major function of the intestinal epithelial barrier is to prevent the passage of bacteria and other pathogens from the lumen into the gut tissue and into systemic circulation, while still allowing passage of nutrients.[13] The gut barrier is maintained by a fine balance of both adherence junctions and tight junctions between epithelial cells, the latter being mainly formed by zonula occludens (ZO) proteins, claudins, and occludins.[13,14] The integrity of these junctions can be compromised by changes to the makeup of the bacteria in the gut[12,15] or by inflammatory changes to the mucosal tissue,[16,17] which can result in the aberrant translocation of luminal contents into the mucosa. For example, bacteria, such as *Collinsella aerofaciens*, which are found in abundance in RA, can increase intestinal permeability in mice through disruption of ZO-1 expression.[18] Similarly, dysregulated mucosal cytokine production, such as increases in interferon γ (IFNγ) or tumor necrosis factor alpha (TNF-α), can reorganize or reduce tight junction protein expression, compromising gut barrier integrity.[19,20] In homeostasis, interleukin-10 (IL-10) expression in the intestinal tissue counteracts transient increases in inflammatory cytokine production and promotes barrier integrity.[16,21] Losses of gut barrier homeostasis have been implicated in the development of a number of systemic diseases, including ankylosing spondylitis (AS),[22–24] type 1 diabetes,[25] and multiple sclerosis.[26,27]

Here, we set out to investigate whether compromised intestinal barrier function is a pathological feature of RA and whether these changes contribute to disease. We report that arthritis is accompanied by loss of gut barrier integrity and altered leukocyte gut homing and that correcting either of these can reduce the severity of arthritis. We show that RA patients have increased serum levels of lipopolysaccharide (LPS), LPS binding protein (LBP), and intestinal fatty acid binding protein (I-FABP), markers of intestinal damage and permeability, and a systemic rise in leukocytes expressing gut-homing markers chemokine receptor 9 (CCR9) and lymphocyte Peyer's patch adhesion molecule 1 (LPAM-1) compared to healthy individuals. We also show that LBP levels were significantly increased in active compared to inactive patients, that they correlated with disease severity, and that loss of gut integrity in RA is partially corrected in etanercept (TNF-α inhibitor)-treated patients. To further understand the impact of arthritis on gut mucosal homeostasis and whether these changes are pathological in disease, we took advantage of spontaneous and induced experimental models of RA. We show that the development of arthritis is intrinsically linked to the development of gut permeability and inflammation. We demonstrate that a loss of gut epithelial cell responsiveness to IL-10 as arthritis develops leads to increased gut permeability and to an exacerbated arthritis. We show, however, that the increase in permeability in arthritis also requires the presence of arthritic cells and gut bacteria.

[1]Centre for Rheumatology, Division of Medicine and Division of Infection and Immunity and Transplantation, University College London, London WC1E 6JF, UK

[2]Evergrande Center for Immunologic Diseases, Harvard Medical School, Boston, MA 02115, USA

[3]Broad Institute of MIT and Harvard, Cambridge, MA 02142, USA

[4]Lydia Becker Institute of Immunology and Inflammation, Division of Infection, Immunity & Respiratory Medicine, University of Manchester, Manchester M13 9PL, UK

[5]Infection, Immunity and Inflammation Programme, UCL Great Ormond Street Institute of Child Health, London WC1N 1EH, UK

[6]Eastman Dental Institute, School of Life and Medical Sciences, UCL, London WC1X 8LD, UK

[7]Centre for Molecular Medicine, Division of Medicine, UCL, London WC1E 6BT, UK

[8]MassGeneral Hospital for Children, Boston, MA 02114, USA

[9]Rheumatology Department, Bicêtre Hospital AP-HP, Université Paris-Saclay and INSERM UMR 1184 IMVA 78 Avenue du Général Leclerc, 94270 Le Kremlin Bicêtre, France

[10]Université de Paris, CNRS, INSERM, UTCBS, Unité des Technologies Chimiques et Biologiques pour la Santé, 75006 Paris, France

[11]Clinical Immunology Laboratory, Groupe Hospitalier Universitaire Paris-Sud, Hôpital Kremlin-Bicêtre, Assistance Publique-Hôpitaux de Paris, 94270 Le-Kremlin-Bicêtre, France

[12]Assistance Publique - Hôpitaux Paris Saclay, Clinical Immunology Laboratory, Hôpital Bicêtre, 94275 Le-Kremlin-Bicêtre, France

[13]Department of Rheumatology, University College London Hospital, London NW1 2BU, UK

[14]Centre for Adolescent Rheumatology Versus Arthritis at UCL, UCLH and GOSH, London WC1E 6JF, UK

[15]These authors contributed equally

[16]Lead contact

*Correspondence: p.blair@ucl.ac.uk (P.A.B.), c.mauri@ucl.ac.uk (C.M.)

https://doi.org/10.1016/j.medj.2021.04.013

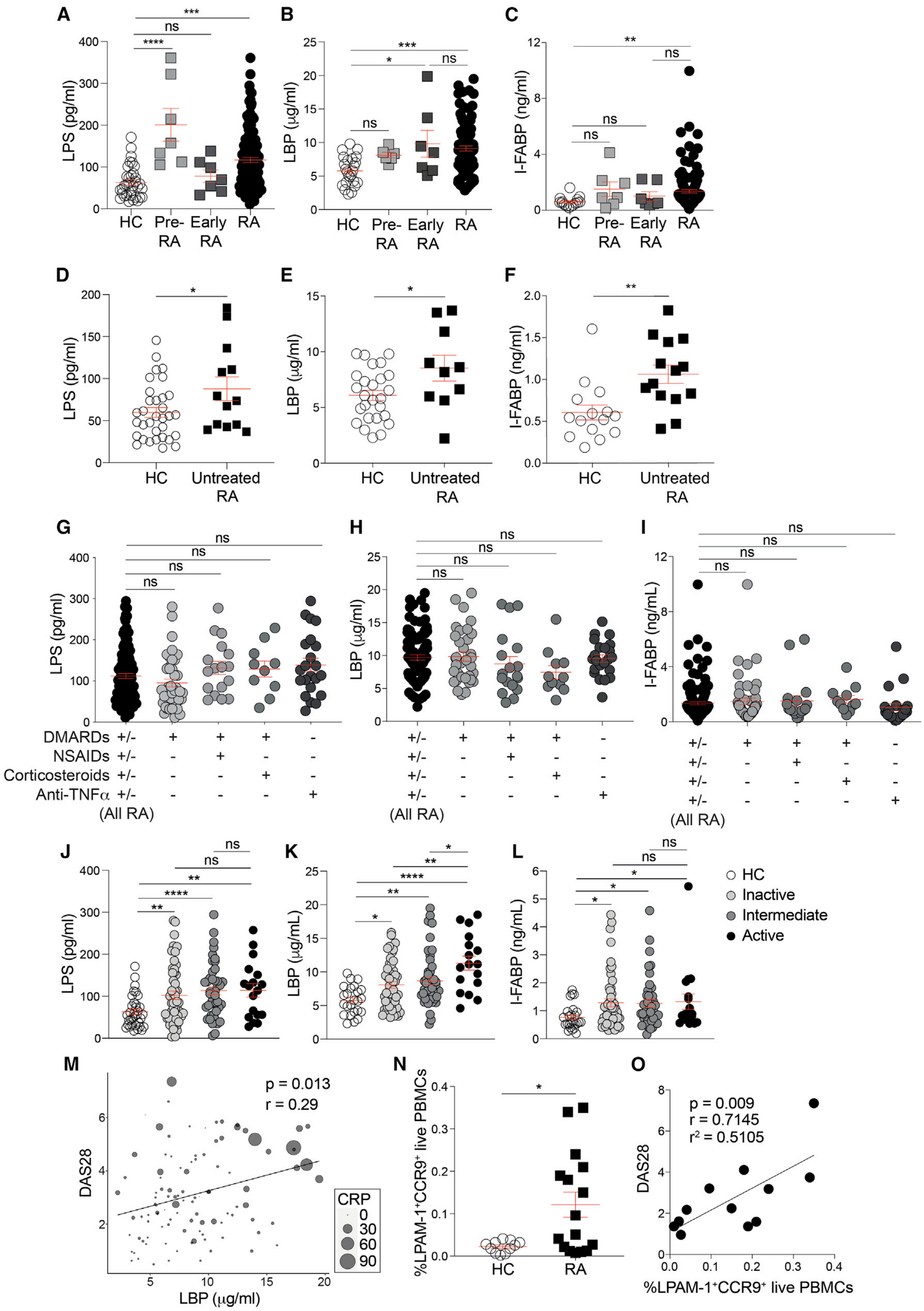
A
LPS (pg/ml)
HC
Pre-RA
Early RA
RA
B
LBP (µg/ml)
C
I-FABP (ng/ml)
D
Untreated RA
E
F
G
DMARDs +/-
NSAIDs +/-
Corticosteroids +/-
Anti-TNFα +/-
(All RA)
H
I
I-FABP (ng/mL)
J
K
LBP (µg/mL)
L
HC
Inactive
Intermediate
Active
M
p = 0.013
r = 0.29
DAS28
LBP (µg/ml)
CRP
0
30
60
90
N
%LPAM-1+CCR9+ live PBMCs
O
p = 0.009
r = 0.7145
r² = 0.5105
ns
*
**
***
****

These findings, together with those showing that constitutively enhanced gut permeability causes worse arthritis and that restoration of tight junction integrity or prevention of recirculation of leukocytes to the gut can ameliorate arthritis, demonstrate the intricacy of the gut-joint axis in the pathogenesis of arthritis.

RESULTS

RA patients display raised levels of serum markers of intestinal barrier damage

To determine whether RA is accompanied by a loss of gut membrane integrity, we compared the serum levels of three biomarkers of gut permeability in RA patients and healthy controls: LPS, a gram-negative bacterial membrane component; LBP, an acute phase protein produced by the intestinal epithelium and the liver in response to translocation of LPS;[28,29] and I-FABP, a specific biomarker of gut epithelial integrity.[30] Patients with serological evidence of arthritis, joint pain but no synovitis (pre-RA), displayed significantly increased levels of LPS, a trend to raised levels of LBP, and moderately elevated I-FABP levels compared to healthy controls, although patients showing early undifferentiated clinical arthritis (early RA) displayed significantly raised levels of LBP and moderately elevated LPS and I-FABP levels compared to healthy controls (HC). All three markers were significantly increased in the serum of RA patients with established disease compared to healthy controls (Figures 1A–1C; Table S1). Common treatments for arthritis, such as non-steroidal anti-inflammatory drugs (NSAIDs), have been reported to increase intestinal permeability.[31] The levels of LPS, LBP, and I-FABP remained significantly higher after exclusion of NSAID-treated patients from the analysis (Figures S1A–S1C). Similarly, the levels of LPS, LBP, and I-FABP were significantly higher in untreated RA patients compared to healthy controls (Figures 1D–1F). Moreover, no differences were observed in levels of gut-permeability markers when patients were stratified according to treatment, ruling out a putative role of NSAIDs in the increase of gut-permeability markers that we observe in this disease (Figures 1G–1I).

To assess whether gut permeability might change according to disease severity, we stratified patients into inactive (28 joint disease activity score [DAS28] < 2.6), intermediate ($2.6 < \text{DAS28} < 5.1$), and active disease (DAS28 > 5.1) and analyzed the levels of serum LPS, LBP, and I-FABP within each group. All activity groups showed significantly higher levels of the three markers than healthy controls. LBP was significantly lower in patients with intermediate or inactive disease compared to patients with active disease. A trend for a reduction in serum LPS concentration in intermediate and inactive disease groups compared to active was also observed (Figures 1J–1L). Analysis of all RA patients revealed that serum LBP concentrations directly

Figure 1. Increased gut permeability in rheumatoid arthritis

(A–C) Concentrations of (A) lipopolysaccharide (LPS), (B) LPS-binding protein (LBP), and (C) intestinal fatty acid binding protein (I-FABP) in the serum of healthy individuals (HC; n = 34), patients with serological evidence of arthritis but no synovitis (pre-RA; n = 7), patients showing early undifferentiated clinical arthritis (early RA; n = 7), and RA patients (n = 133) by ELISA.

(D–F) Concentrations of (D) LPS, (E) LBP, and (F) I-FABP in the serum of healthy individuals (n = 34) and untreated RA patients (n = 14 LPS and I-FABP; n = 10 LBP).

(G–I) Concentrations of (G) LPS, (H) LBP, and (I) I-FABP in serum from RA patients stratified by disease severity (remission—DAS28 < 2.6, intermediate—DAS28 > 2.6 and <5.1, and active—DAS28 > 5.1), measured by ELISA.

(J–L) Concentrations of (J) LPS, (K) LBP, and (L) I-FABP in serum from RA patients stratified by treatment group, measured by ELISA.

(M) Correlation between LBP and disease activity scores measured by DAS28 (n = 124). Circle sizes reflect CRP level of each patient.

(N) Flow cytometric analysis of the expression of LPAM-1 and CCR9 on live peripheral blood mononuclear cells (PBMCs) from RA patients (n = 16) and healthy controls (n = 11).

(O) Correlation between LPAM-1^{+}CCR9^{+} live PBMCs and disease activity scores measured by DAS28 (n = 13).

*$p < 0.05$; **$p < 0.01$; ***$p < 0.001$; ns, no significance. (A–C and G–I) One-way ANOVA with multiple comparisons calculated by Tukey's test, (D–F and N) unpaired t test, (J–L) Mann-Whitney t test, and (M and O) Pearson's correlations. Data represent mean ± S.E.M.

correlate with both DAS28 and C-reactive protein (CRP), with the highest combined DAS28 scores and CRP levels recorded in patients with the highest levels of serum LBP (Figures 1M and S1D).

Stratification of our cross-sectional cohort of RA patients showed that the levels of gut-permeability markers were not affected by the different treatments. However, patients with less active disease displayed lower levels of LBP and a trend for a reduction in the levels of LPS, suggesting that inhibition of inflammation in RA by successful treatment may be able to reduce gut permeability. Thus, given that some TNF-α inhibitors can be effective treatments for both RA and inflammatory bowel diseases, it was surprising that we observed no effect in treatment stratification. To address whether neutralization of TNF-α affects the levels of serum markers of gut permeability, we tested the concentration of LPS, LBP, and I-FABP in the serum of a longitudinal cohort of TNF-α inhibitor (etanercept)-treated RA patients before and 6 months after the start of treatment (6 months is a sufficient time to determine response to therapy; Figures S1E–S1G). Patients responding to etanercept therapy, defined as a decrease in DAS28 of more than 1.2, showed significant reductions in levels of serum LPS and LBP. Levels of I-FABP were, however, unaffected by etanercept. Patients who did not respond to etanercept treatment did not display a reduction in any of the markers of gut permeability.

It has been previously shown that CCR9 and LPAM-1 are expressed predominantly by lymphocytes that recirculate to and from the gut. Mice deficient in CCR9 or vitamin A, the dominant inducer of CCR9 and LPAM-1 expression, have reduced numbers of lymphocytes recruited to the intestinal lamina propria.[32,33] RA patients displayed an increase in the percentage of PBMCs co-expressing the gut-tropic markers LPAM-1 and CCR9 (Figures 1N and S1H), suggesting an increase in gut homing in RA. The percentage of LPAM-1^{+}CCR9^{+} PBMCs also positively correlated with disease severity (DAS28), further linking immune events in the gut with the pathogenesis of RA (Figure 1O).

Arthritic mice display increased intestinal permeability and inflammation

To investigate in detail how the integrity of the gut is affected during the development of arthritis, we took advantage of the K/BxN spontaneous mouse model of arthritis, which mimics several aspects of RA (Figure S2A).[34] For clarity of presentation, we define different stages of disease as pre-disease (at 2 weeks), early disease (at 4 weeks), and established disease (at 6 weeks). Control mice (CTRL) were 6-week-old naive C57BL/6 mice. Increased levels of serum LBP were observed in mice at early stages of disease and in mice with established arthritis compared to CTRL and pre-disease mice (Figure 2A). Serum LBP concentration positively correlated with the severity of joint swelling (Figure 2B). Further assessment of gut barrier integrity by measuring uptake of orally gavaged fluorescein isothiocyanate (FITC)-dextran confirmed an increase in permeability from an early stage of arthritis, rising to a peak at the height of disease (Figure 2C). Histologically, we observed a loss of expression of ZO-1, a critical mediator of tight junction assembly and integrity,[35,36] by the small intestinal (SI) and colonic epithelial cells of arthritic mice (Figures 2D, 2E, S2B, and S2C). In agreement with serum measurements of permeability, ZO-1 expression also decreased from the earliest stages of disease. In addition, significant morphological changes, including epithelial erosion and crypt elongation, were detected in the SI (Figures 2F and 2G) and colon (Figures S2D and S2E) of K/BxN mice with early and established arthritis, compared to pre-disease and CTRL mice.[37] Of note, the presence of inflammatory cell infiltrates in the SI and the colons of arthritic mice suggested immune-cell-mediated alterations to the intestines (Figures 2F, 2G, S2D, and

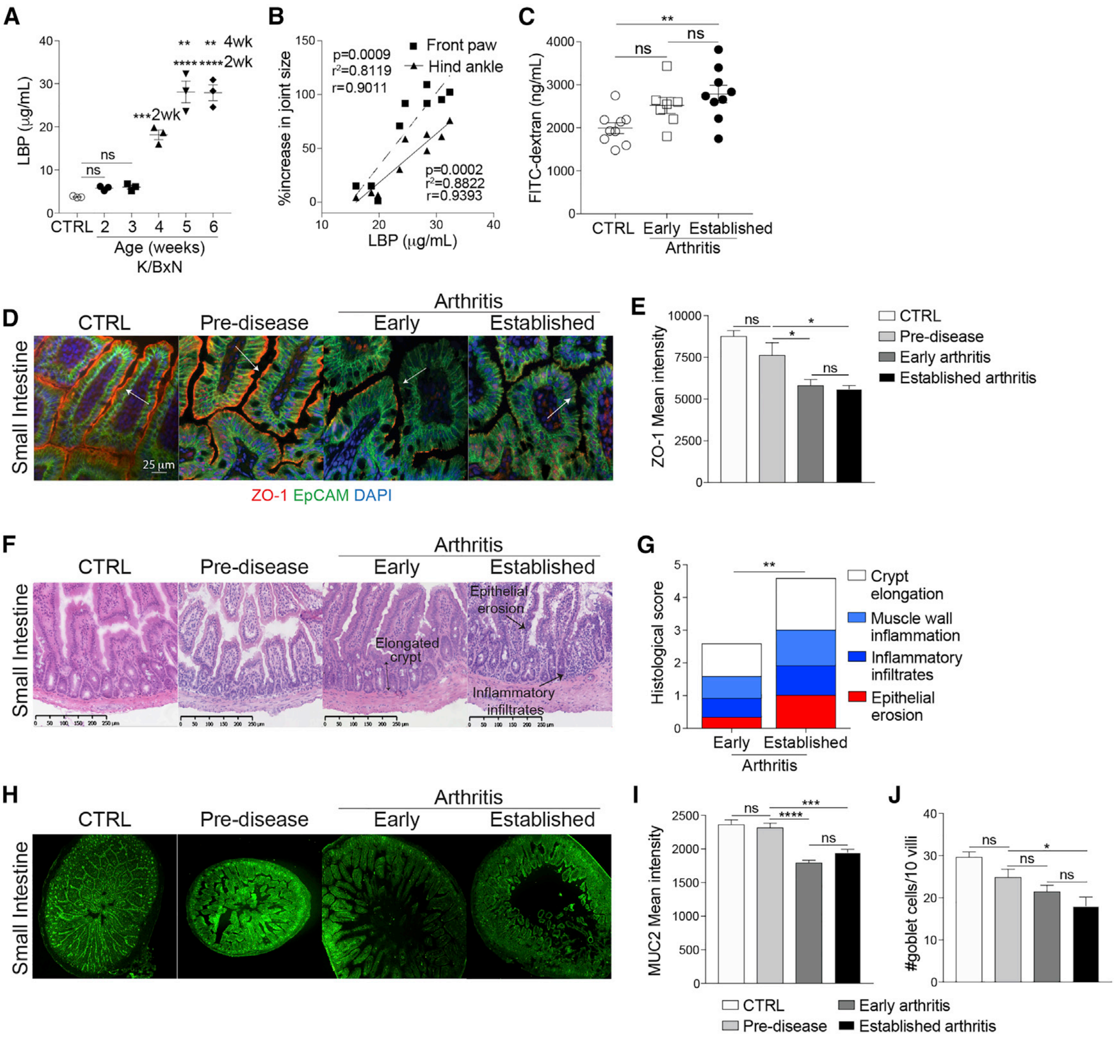

Figure 2. Mice with arthritis display intestinal permeability and intestinal inflammation

(A) Concentration of LBP in serum of K/BxN mice at marked ages and naive C57BL/6 control mice (n = 3/group), as measured by ELISA.

(B) Correlation of serum LBP concentration with increase in joint size in 4- to 5-week-old K/BxN mice (n = 8).

(C) Concentration of FITC-dextran in serum of naive C57BL/6 control mice (CTRL) (n = 9) and K/BxN mice with early arthritis (n = 7) and with established arthritis (n = 9).

(D–J) Representative (D) ZO-1 staining, (E) mean ZO-1 intensity, (F) H&E staining, (G) histological scores, (H) MUC2 staining, (I) mean MUC2 intensity, and (J) goblet cell counts from periodic acid-Schiff (PAS) staining for SI of K/BxN mice throughout arthritis development and compared to naive C57BL/6 control mice.

*$p < 0.05$; **$p < 0.01$; ***$p < 0.001$; ****$p < 0.0001$. (A, C, E, I, and J) One-way ANOVA, (B) Pearson's correlations, and (G) two-way ANOVA. For all panels, one out of two experiments is shown. Data represent mean ± S.E.M.

S2E). In contrast to the morphological changes and loss of ZO-1 expression observed in the intestinal epithelium of arthritic mice, we detected no significant changes in the mucosal epithelium of the lung, suggesting that the loss of mucosal epithelial integrity is primarily restricted to the gut (Figures S2F–S2H).

The integrity of the mucous-layer lining of the luminal side of the epithelium is an objective measurement of gut health and homeostasis,[15] with mucin 2 (MUC2) being the most abundant protein component of the intestinal mucous layer.[38] We report a significant loss of MUC2 in the SI of arthritic K/BxN mice compared to pre-disease and CTRL mice (Figures 2H and 2I). Goblet cell numbers were also significantly

reduced on the villi of the SI of arthritic mice compared to CTRL, supporting the reduction in MUC2 expression (Figure 2J). No differences in MUC2 protein levels were detected in the colon of arthritic mice compared to pre-disease and CTRL mice, despite an increase in goblet cell numbers in arthritis (Figures S2I–S2K).

A combination of arthritic microbiota and cells drive disruption of gut permeability

Given the accumulation of inflammatory infiltrates we observed in arthritic mice and considering the role of the microbiota in the development of arthritis both in humans and in mouse models,[2,39] we next investigated the contributions of peripheral arthritic lymphocytes and gut bacteria to the loss of intestinal barrier integrity. In agreement with patient data showing higher LPAM-1$^+$CCR9$^+$ expression by RA PBMCs compared to healthy individuals (Figure 1N), we observed significant increases in the frequency of LPAM-1$^+$CCR9$^+$CD45$^+$ cells in the spleens, Peyer's patches (PPs), mesenteric lymph nodes (mLNs), and paw-draining axillary lymph nodes (ALNs) in arthritic K/BxN mice compared to CTRL mice (naive non-obese diabetic [NOD] mice), suggesting that increased cellular circulation to the gut is a feature of arthritis (Figure 3A).

To address directly whether cells from arthritic mice, either alone or in combination with arthritic gut microbiota, contribute to the loss of gut barrier integrity, we transferred arthritic splenocytes and/or feces to lymphocyte-deficient NOD *scid* gamma (NSG) mice and assessed the effects on gut permeability by FITC-dextran uptake. Arthritic splenocytes showed an elevated expression of pro-inflammatory cytokines, such as IFNγ, IL-6, and TNF-α (Figure 3B). Feces from arthritic K/BxN donor mice showed an enrichment of bacteria previously published to be associated with arthritis,[4–7] including an expansion of bacteria from the *Prevotella* genus, compared to pre-disease mice and to the KRN control (Figures 3C, S2L, and S2M). To remove potential confounding effects of endogenous bacteria, prior to cell or fecal transfer, NSG mice were treated with broad-spectrum antibiotics known to ablate gut bacteria.[40] NSG recipients were then orally gavaged with feces from arthritic K/BxN mice or control KRN mice (the maternal strain for K/BxN mice) and left to reconstitute for 1 week. Arthritic or non-arthritic control splenocytes (splenocytes from NOD mice, the paternal strain for K/BxN mice) were transferred intravenously to untreated, antibiotics-treated, or fecal-transfer-recipient NSG mice, and intestinal permeability was measured 2 days later. Reconstitution of antibiotics-treated NSG mice with feces alone from either control or arthritic K/BxN mice did not significantly increase permeability compared to untreated NSG mice (Figures 3D and 3E). Transfer of control splenocytes, with or without fecal gavage, did not increase permeability (Figure 3E). Transfer of splenocytes from arthritic K/BxN mice resulted in a moderate increase in permeability in untreated NSG mice. However, it was only when K/BxN splenocytes were co-transferred with K/BxN feces that we noted a significant increase in permeability compared to untreated NSG mice (Figure 3D). These results demonstrate that both arthritogenic cells and arthritogenic gut microbiota are necessary to induce disruption of gut permeability.

One of the consequences of increased gut permeability may be the translocation of the bacteria themselves into circulation. Mice with established arthritis displayed an accumulation of bacterial 16 s DNA in mLNs, ALNs, and in spleens compared to mice in the early stage of disease (Figure 3F). These findings further confirm that arthritis development is accompanied by a loss of intestinal barrier integrity and also show that this defect leads to a systematic dissemination of bacteria, which could contribute to the pathogenesis of arthritis.

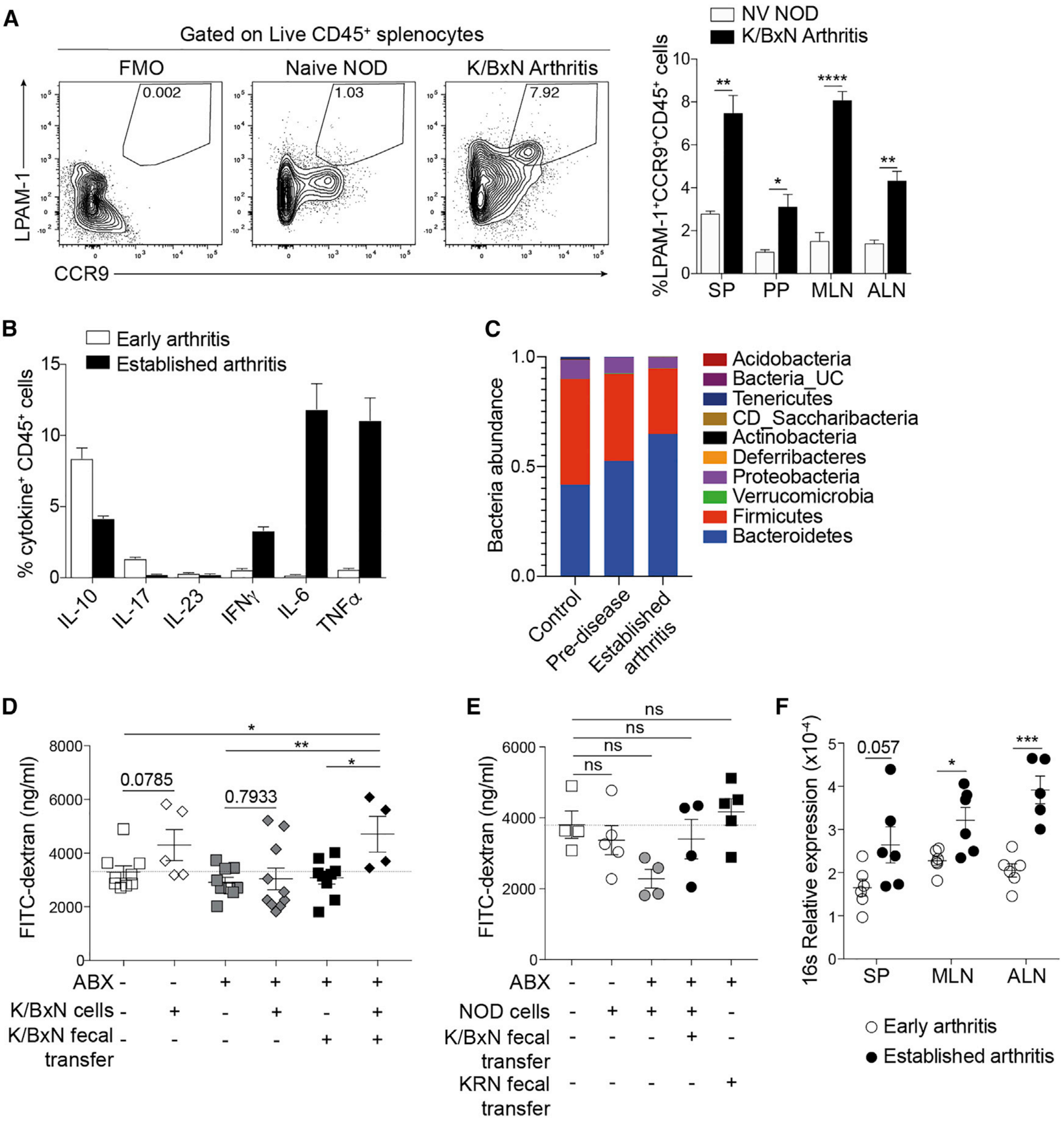

Figure 3. Arthritic splenocytes and dysbiosis can cause increased intestinal permeability

(A) Representative fluorescence-activated cell sorting (FACS) plots of LPAM-1 and CCR9 expression by splenocytes and bar chart showing the mean percentage expression of LPAM-1^+CCR9^+ on total CD45^+ isolated from noted tissues from K/BxN mice with established arthritis (n = 4) and naive NOD controls (n = 3).

(B) Mean percentage of CD45^+ splenocytes isolated from K/BxN mice in the early stages of disease and with established arthritis (n = 5/group) expressing marked cytokines.

(C) Bar chart showing the abundance of the noted bacterial phyla in samples collected from K/BxN mice at pre-disease (3 weeks old) and established arthritis (6 weeks old), compared to control (KRN maternal strain).

(D) Concentration of FITC-dextran in serum of untreated or antibiotics-treated NSG mice, with or without reconstitution with feces and/or splenocytes from arthritic K/BxN mice (n $\geq$ 4/group).

(E) Concentration of FITC-dextran in serum of untreated or antibiotics-treated NSG mice, with or without reconstitution with feces from arthritic K/BxN mice or KRN control, and with or without transfer of splenocytes from control NOD donors (n $\geq$ 4/group).

(F) Degree of bacterial translocation to the spleen (SP), mesenteric (MLN), and joint-draining (axillary; ALN) lymph nodes of K/BxN mice in the early stages of disease and with established arthritis, as assessed by levels of bacterial 16 s rDNA measured by qPCR (n = 6/group).

*$p < 0.05$; **$p < 0.01$; ***$p < 0.001$; ****$p < 0.0001$. (A, D, and F) Unpaired t test and (E) one-way ANOVA with multiple comparisons calculated by Tukey's test. For all panels, one out of two experiments is shown. Data represent mean ± S.E.M.

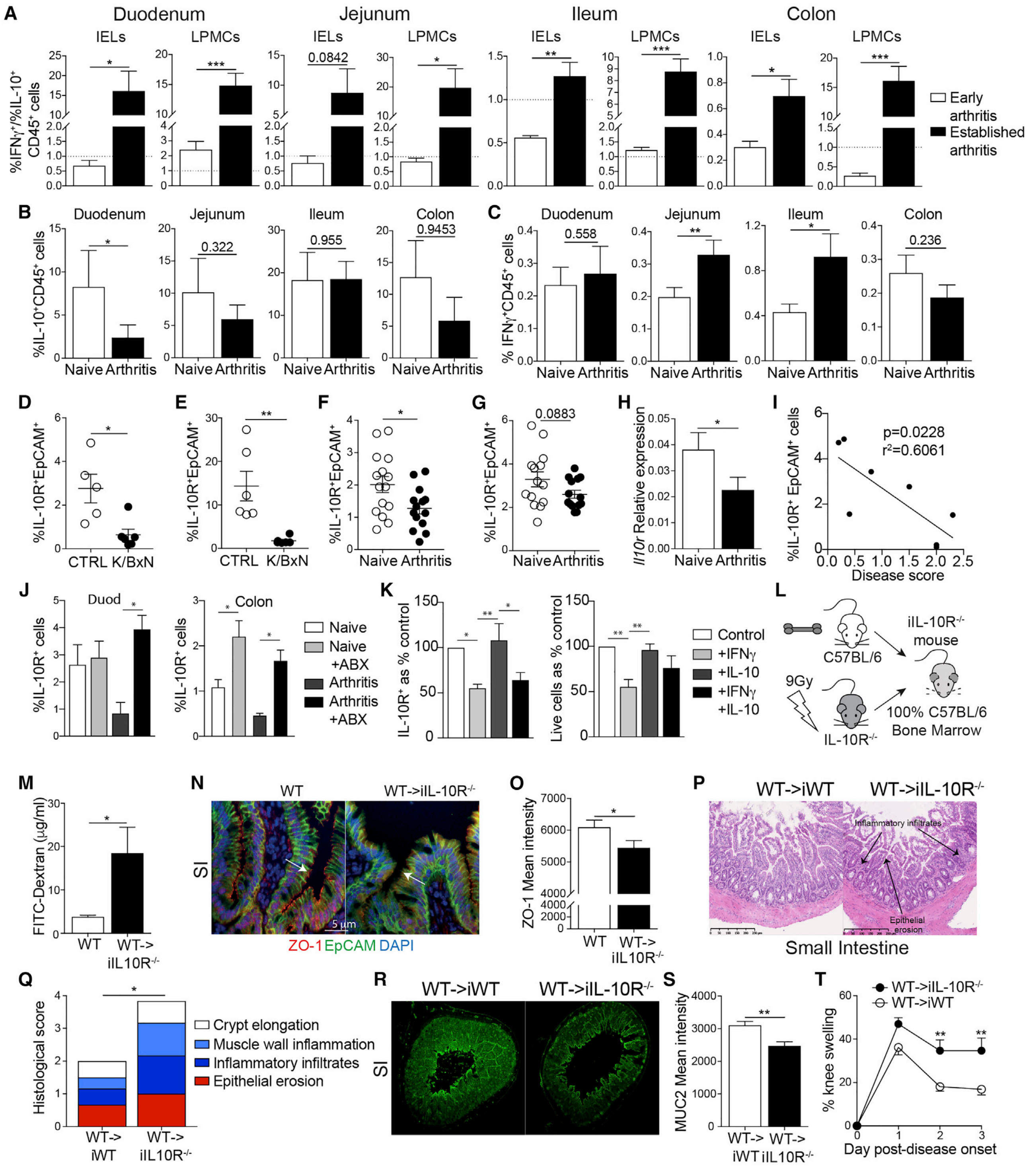

Figure 4. Loss of IL-10 signaling affects intestinal permeability and arthritis severity

(A) Bar graphs displaying the ratio of IFNγ$^+$CD45$^+$ cells to IL-10$^+$CD45$^+$ cells from intra-epithelial lymphocytes (IELs) and lamina propria mononuclear cells (LPMCs) isolated from early-arthritic K/BxN mice (n = 5) and K/BxN mice with established arthritis (n = 6), as assessed by flow cytometry.

(B and C) Mean percentages of (B) IL-10$^+$CD45$^+$ and (C) IFNγ$^+$CD45$^+$ IELs isolated from C57BL/6 naive and arthritic mice (AIA day 3; n = 8/group), as assessed by flow cytometry.

(D–G) Mean percentage expression of IL-10R by intestinal epithelial cells isolated from the (D) duodenum and (E) colon of naive C57BL/6 CTRL and K/BxN mice with established arthritis (n = 6/group) and (F) duodenum and (G) colon of naive C57BL/6 and arthritic mice (AIA day 3; n = 14/group).

Loss of intestinal epithelial IL-10 signaling contributes to gut barrier dysfunction and arthritis severity

To confirm our results in a second arthritis model and to determine whether the increase in gut permeability associated with arthritis would normalize as disease abates, we took advantage of a self-remitting antigen-induced arthritis (AIA) mouse model (Figure S3A).[41,42] Mice in the acute phase of arthritis showed increased levels of serum LBP and FITC-dextran and decreased epithelial ZO-1 expression compared to naive mice (Figures S3B–S3H). Mice with AIA displayed similar inflammatory morphological changes to the gut mucosa as arthritic K/BxN mice and a systemic increase in LPAM-1 and CCR9 expression during disease (Figures S3I–S3N). Of interest, both gut inflammation and intestinal barrier integrity, as well as expression of gut homing markers, were partially recovered during the remission phase of AIA, suggesting that inflammation and/or activation of the peripheral immune system is intimately linked with changes to the health and permeability of the gut (Figures S3B and S3E–S3N). Similarly to the K/BxN model, morphological changes to the mucosal epithelium and loss of ZO-1 expression were not observed in the epithelium of the lung (Figures S3O–S3Q). Mice with AIA also displayed dysbiosis of the intestinal microbiota, with a significant increase in abundance of *Prevotella* and a trend in reduction of *Lactobacillus* (Figures S3R–S3T). These changes in bacterial abundance were apparent from day 1 of disease but were more pronounced at the peak of arthritis (Figures S3R–S3T).

Given that we observed an increase in inflammatory infiltrates in the gut mucosa of arthritic mice and that recirculating pro-arthritogenic cells are implicated in the loss of gut barrier integrity, we next assessed whether morphological changes to the gut in arthritis were accompanied by alterations in the balance of pro- and anti-inflammatory cytokines produced by $CD45^+$ leukocytes in the intestines. Arthritic K/BxN mice displayed a significantly higher ratio of $IFN\gamma^+$ to $IL\text{-}10^+CD45^+$ leukocytes in the duodenum, jejunum, ileum, and colon compared to mice in the early stages of disease (Figures 4A and S4A–S4C). Mice with AIA displayed a similar loss of IL-10 expression to K/BxN mice and a gain in IFNγ expression by $CD45^+$ cells in the intestinal epithelium (Figures 4B and 4C). As with changes to the integrity of the mucosal epithelium, leukocytes in the lung tissue did not display the same degree of inflammatory changes as those in the intestines (Figures S4D and S4E). Although there was a trend for minor increases in the frequency of $IFN\gamma^+$ cells in the lungs of K/BxN mice compared to naive controls, mice with AIA displayed no difference to their controls.

Several reports have suggested that IL-10-IL-10R signaling is important for maintaining gut homeostasis and protecting the epithelium from inflammatory damage.[43–45]

(H) Relative expression of *il10r*, as measured by qPCR, of intestinal epithelial cells from (F) (n = 6).
(I) Correlation between duodenal epithelial cell IL-10R expression and disease activity, as measured by knee swelling in C57BL/6 arthritic mice (AIA day 3; n = 8).
(J) IL-10R expression by epithelial cells isolated from the duodenum (duod) and colon of untreated and antibiotics-treated (+ABX) naive mice and mice with AIA (n = 4).
(K) Mean percentages of live epithelial cells and mean percentages of IL-10R expression by live epithelial cells, following culture of SI-derived organoids with IFNγ, IL-10, or both. Percentages normalized to control organoid expression (no IL-10/IFNγ; n = 4).
(L) Schematic showing the generation of bone marrow chimera lacking IL-10R on non-hematopoietic cells.
(M) Levels of FITC-dextran in serum of naive WT or WT→iIL-$10R^{-/-}$ mice.
(N and O) Representative ZO-1 staining and mean ZO-1 intensity of SI sections from naive WT or WT→iIL-$10R^{-/-}$ mice.
(P–T) AIA was induced in WT→iIL-$10R^{-/-}$ and WT→iWT (chimeric controls that express IL-10R) mice. At day 3 of arthritis, mice were sacrificed.
(P and Q) Representative H&E staining and histological scores of the SI.
(R and S) Representative MUC2 staining and mean MUC2 intensity of the SI.
(T) Mean clinical score of arthritis (n = 3 and 4, respectively).
*$p < 0.05$; **$p < 0.01$; ***$p < 0.001$. (A, D–H, M, O, and S) Unpaired t test, (B and C) paired t test, (I) Pearson's correlations, (J and K) one-way ANOVA, and (Q and T) two-way ANOVA. For all panels, one out of two experiments is shown. Data represent mean ± S.E.M.

Ex vivo, both arthritic K/BxN and C57BL/6 mice with AIA showed significant reductions of IL-10R expression by the intestinal epithelial cells (IECs) of the duodenum and colon compared to their respective CTRLs (Figures 4D–4H, S5A, and S5B). Further, *ex vivo* IL-10R expression by the duodenal IECs displayed a significant inverse correlation with the degree of arthritis severity (as measured by knee swelling on day 3 of AIA), reinforcing the link between arthritis severity and loss of IL-10R expression by IECs (Figure 4I).

Next, we investigated whether the loss of IL-10R expression by IECs could be a consequence of arthritis-related dysbiosis and/or the result of the elevated IFNγ expressed by intra-epithelial lymphocytes (IELs) and lamina propria mononuclear cells (LPMCs). To assess the role of gut microbiota in the loss of IEC IL-10R expression, arthritic mice and naive controls were treated with a cocktail of antibiotics to remove bacteria. The inhibition of IL-10R expression observed in arthritic mice was corrected by antibiotics treatment, with levels of IL-10R expression restored to those seen in naive mice in both the duodenum and colon (Figure 4J). To assess the role of IFNγ in the inhibition of IEC IL-10R expression, organoids, grown from crypts isolated from the small intestines of naive C57BL/6 mice, were cultured with IFNγ, IL-10, or in combination. At the end of culture, epithelial cell IL-10R expression was measured by flow cytometry (Figures 4K and S5C–S5E). The expression of IL-10R by organoid IECs, as well as IEC viability, were both reduced after culture with exogenous IFNγ. Organoid IEC survival was partially rescued by the addition of IL-10. Taken together, these results suggest that both arthritis-associated bacteria and inflammatory cytokines, such as IFNγ, contribute to the loss of IEC IL-10R expression in arthritis.

To understand the role that IEC IL-10R expression plays in the maintenance of gut barrier integrity in arthritis, we generated chimeric mice that lacked IL-10R on the non-hematopoietic cells but retained expression on cells of the immune system (wild type [WT]→irradiated IL-10R$^{-/-}$ [WT→iIL-10R$^{-/-}$] mice; Figure 4L). A significant increase in FITC-dextran translocation and a loss of ZO-1 expression on gut epithelial cells of both the SI and colon were observed in naive WT→iIL-10R$^{-/-}$ mice compared to control WT mice, similar to the loss of ZO-1 in the SI of naive global IL-10R$^{-/-}$ mice compared to control, demonstrating that the expression of IL-10R on non-hematopoietic cells contributes directly to the maintenance of gut permeability (Figures 4M–4O and S5F–S5I). Upon AIA induction, WT→iIL-10R$^{-/-}$ mice displayed exacerbated intestinal inflammation, including a significant loss of the mucous layer in the SI compared to arthritic WT→iWT mice (Figures 4P–4S, S5J, and S5K). WT→iIL-10R$^{-/-}$ mice also developed significantly worse arthritis compared to arthritic WT→iWT mice (Figure 4T). Thus, our results suggest that the impairment in IL-10R expression on gut epithelial cells contributes to the disruption of gut barrier integrity and to increased joint inflammation.

A constitutive increase in intestinal permeability predisposes mice to more severe arthritis

To investigate whether the changes in joint swelling observed in WT→iIL-10R$^{-/-}$ mice are directly linked to increased gut permeability, rather than the wider loss of IL-10R expression or immune dysregulation, we took advantage of a recently generated but uncharacterized claudin-8$^{-/-}$ mouse (*Cl8*$^{-/-}$) . Claudin-8 is a tight junction protein that decreases paracellular permeability, and lack of claudin-8 expression has been previously associated with Crohn's disease and gut barrier dysfunction.[46,47] Compared to WT mice, naive *Cl8*$^{-/-}$ mice present a constitutively increased intestinal permeability (Figure 5A). Upon AIA induction, *Cl8*$^{-/-}$ mice develop a significantly exacerbated arthritis

CellPress
OPEN ACCESS

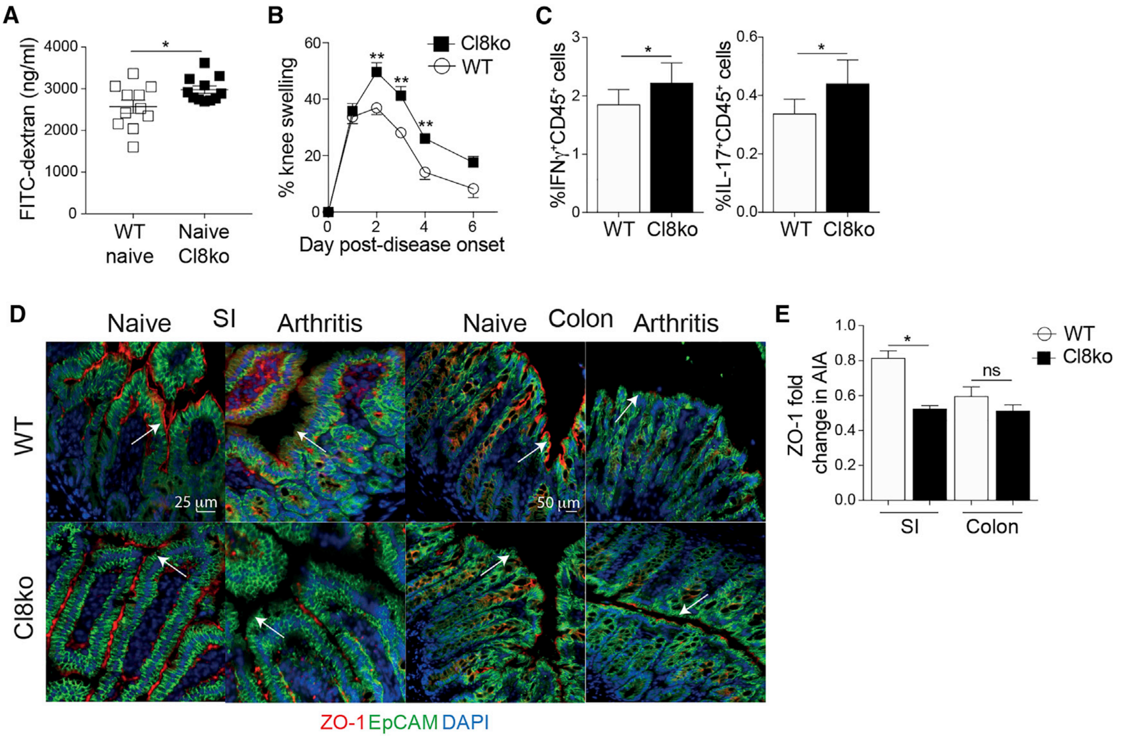

Figure 5. Claudin8⁻/⁻ mice have increased gut permeability and develop more severe arthritis

(A) FITC-dextran concentration in the serum of naive WT and claudin8$^{-/-}$ (Cl8ko) mice (n = 11).

(B) Mean clinical score of arthritis in WT and claudin8$^{-/-}$ mice (n = 7/group).

(C) Mean percentage expression of splenic IFNγ and IL-17 by total CD45^{+} cells from WT and claudin8$^{-/-}$ mice at day 7 of AIA (n = 7/group).

(D and E) Representative ZO-1 staining and mean ZO-1 intensity in the SI and colon of WT and claudin8$^{-/-}$ mice on day 3 of AIA, compared to their respective naive controls.

*$p < 0.05$; **$p < 0.01$. (A, C, and E) Unpaired t test and (B) two-way ANOVA. For all panels, one out of two experiments is shown. Data represent mean ± S.E.M.

compared to WT controls (Figure 5B) and a rise in the frequencies of IFNγ^{+} and IL-17^{+}CD45^{+} cells in the spleen (Figure 5C). The increase in severity of joint inflammation was accompanied by a loss of ZO-1 expression by intestinal epithelial cells compared to arthritic WT mice (Figures 5D and 5E). These data suggest that enhanced gut permeability predisposes mice to develop a more severe joint swelling.

Modulating gut permeability and cellular recirculation ameliorates arthritis

The above results prompted us to investigate whether preventing the loss of intestinal barrier integrity could attenuate arthritis. To assess this, we used a small molecule zonulin antagonist, AT-1001, that prevents zonulin-mediated retraction of tight junctions.[48] AT-1001 is of particular interest as it is currently being tested for its therapeutic effect in celiac disease and could be repurposed to a rheumatological setting.[49] Treatment of arthritic mice with AT-1001 by oral gavage (Figure S6) prevented the disruption of gut permeability, as shown by significantly reduced FITC-dextran uptake compared to untreated mice (Figure 6A) and dramatically reduced joint swelling (Figure 6B). This was accompanied by a significant reduction in systemic and intestinal inflammation as shown by the decreased frequency of IFNγ^{+} and IL-17^{+}CD45^{+} splenocytes (Figure 6C) and by the lower levels of intestinal tissue damage, including crypt elongation and epithelial erosion (Figures 6D, 6E, S6B, and S6C). MUC2 expression was also increased in the intestines of AT-1001-treated mice compared to untreated arthritic mice, confirming the reduction in mucosal inflammation (Figures 6F and 6G). AT-1001 treatment also partially prevented the loss of

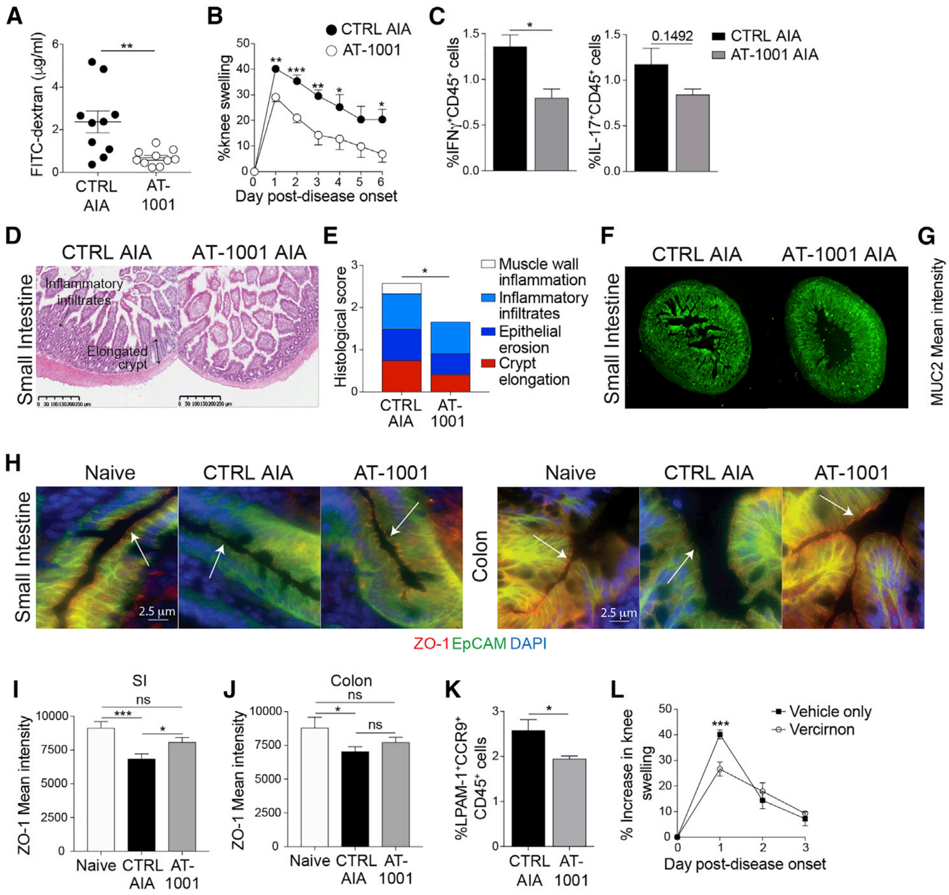

Figure 6. Preventing the loss of gut barrier integrity decreases arthritis severity

(A) FITC-dextran concentration in the serum of untreated and AT-1001-treated C57BL/6 mice on day 3 of AIA (n = 10/group).

(B) Mean clinical score of untreated and AT-1001-treated mice following induction of AIA (n = 5/group).

(C) Mean percentage expression of splenic IFNγ$^+$ and IL-17$^+$ total CD45$^+$ cells from untreated and AT-1001-treated arthritic mice (n = 5/group).

(D–G) Representative (D) H&E staining, (E) histological scores, (F) MUC2 staining, and (G) mean MUC2 intensity in the small intestines of untreated and AT-1001-treated arthritic mice (day 3 of AIA).

(H–J) Representative ZO-1 staining and mean ZO-1 intensity in SI and colon of naive mice and untreated and AT-1001-treated arthritic mice (day 3 of AIA).

(K) Mean percentage expression of LPAM-1$^+$ and CCR9$^+$ on total CD45$^+$ splenocytes from untreated and AT-1001-treated arthritic mice (n = 5/group).

(L) Mean clinical score of arthritis in vehicle control and vercirnon-treated C57BL/6 mice (n = 5/group).

*$p < 0.05$; **$p < 0.01$; ***$p < 0.001$. (A, C, and K) Unpaired t test, (G, I, and J) one-way ANOVA, and (B, E, and L) two-way ANOVA. For (A)–(K), one out of two experiments is shown. Data represent mean ± S.E.M.

ZO-1 expression on the intestinal epithelial cells that is observed during arthritis (Figures 6H–6J).

Another consequence of AT-1001 treatment is a reduction in the percentage of LPAM-1$^+$CCR9$^+$CD45$^+$ cells in the spleens of mice with AIA compared to untreated mice (Figure 6K). These results, along with the findings that the percentages of LPAM-1$^+$CCR9$^+$ cells are increased in both the PBMCs of RA patients (Figure 1N) and systemically in arthritic K/BxN and AIA mice (Figures 3A and S3N), prompted us to assess whether inhibiting the recirculation of these cells between the gut and the periphery would reduce the severity of arthritis. Blocking CCR9-mediated recirculation, using the specific CCR9 antagonist vercirnon,[50] significantly reduced joint swelling during the peak of disease in mice with AIA compared to untreated controls (Figure 6L).

DISCUSSION

In the present study, we explore two critical aspects of the contribution of the gut to the development of RA, the loss of intestinal epithelial barrier integrity and the development of subclinical gut inflammation. Previous studies have suggested that enhanced intestinal permeability may be present in arthritis; however, these studies did not rule out drug effects, nor did they show a link to the severity of disease. Bjarnason et al.[51] and Mielants et al.[23,31] used ^{51}Cr-EDTA absorption assays to investigate gut "leakiness" in RA, AS, and osteoarthritis. Although they found increased uptake of ^{51}Cr-EDTA, and thus increased gut permeability, in all three diseases, they concluded that the increased uptake in RA was a result of NSAID treatment rather than disease. A more recent study by Tajik et al.[52] demonstrated that loss of gut epithelial integrity could be detected in both new onset and established RA patients but did not investigate the role of treatment in either the evolution or resolution of gut permeability. Here, we show that serum LPS, LBP, and I-FABP levels, markers of gut damage and permeability, are raised in active RA patients compared to healthy controls and that these measures are independent from the treatment, implying that enhanced permeability is a feature of disease rather than of therapy. We also report a positive correlation between the severity of disease and the levels of LBP.

Interestingly, analysis of gut-permeability markers in a longitudinal cohort of RA patients treated with the TNF inhibitor etanercept showed a partial restoration of gut integrity in patients responding to the treatment. The decreased levels of gut-permeability markers are likely a consequence of reduced systemic inflammation, which may lead to both a reduction in the recruitment of pro-inflammatory cells to the gut, as well as a reduction in the production of intestinal inflammatory cytokines (as discussed below in more detail in relation to the role of IFNγ in enhancing gut permeability). However, the results showing that etanercept could not completely restore gut integrity suggest that additional mechanisms contribute to this loss in arthritis. For example, dysbiosis can directly contribute to the increase of gut permeability,[18] and it has been previously reported that etanercept can only partially correct dysbiosis in RA patients.[53] Increased permeability in RA patients may also be due to genetic predispositions, such as polymorphisms in zonulin or myosin IXB (MYO9B) that have been associated with loss of gut integrity in inflammatory bowel disease (IBD).[54,55] Arthritis-induced gut damage may, however, also take longer than the period of our study to resolve. A number of TNF inhibitors are currently licensed for the treatment of RA and IBD,[56,57] and a comparison of their ability to correct intestinal inflammation and permeability, as well as RA symptoms, would be warranted.

Early direct evidence for subclinical gut inflammation in RA showed abnormal focal accumulations of ^{111}indium radio-labeled leukocytes in the intestines of 12 of 26 tested RA patients.[58] Electron microscopy of biopsies of the ileums and colons of RA patients suggested that ultrastructural changes, such as destruction of the brush border and a loss of microvilli density, could be detected in a subset of tested patients, though these changes were less evident by light microscopy or endoscopy and were not as obvious as in biopsies of spondyloarthropathy patients.[59] By taking advantage of two different experimental models of arthritis, one spontaneous and one induced, we show increased intestinal permeability as measured by FITC-dextran translocation and loss of tight junction protein ZO-1, as well as extensive inflammatory changes to the intestinal epithelium, muscle wall, and inflammatory infiltrates during arthritis from the earliest stages of disease. These morphological changes were matched by an infiltration of IFNγ-expressing inflammatory

leukocytes and a loss of anti-inflammatory IL-10-expressing cells, supporting previous preliminary human studies suggesting that the gut is a site of leukocyte infiltration during arthritis.[58] Of interest, despite the fact that up to 60% of patients with RA can develop pathological changes in the lung, we did not detect any significant changes to the mucosa of the lungs, at least in these preclinical models.

Previous studies in RA patients have reported tight-junction-disrupting bacteria, such as *Collinsella aerofaciens* and *Porphyromonas gingivalis*, to be associated with disease,[18,60] suggesting a role for bacteria in driving gut permeability. Our data also support a pivotal role of arthritogenic bacteria in driving alteration of gut barrier. However, they have also added a degree of complexity, as we showed that both the transfer of arthritogenic feces and splenocytes, expressing high levels of IFNγ, TNF-α, and IL-6, and low levels of IL-10 were required to induce permeability in NSG immune-deficient recipient mice. This result lends support to previous *in vitro* findings, using colonic epithelial cell lines, showing that the inflammatory cytokines IFNγ and TNF-α cooperate to induce epithelial barrier dysfunction by causing epithelial cell ZO-1 internalization, downregulation of occludin, and cell death.[16,17,19,20] A number of mechanisms by which increased intestinal permeability could contribute to autoimmunity have been proposed. In type 1 diabetes, it has been suggested that microbial translocation to the pancreatic lymph nodes results in increased nucleotide-binding oligomerization domain containing protein 2 (NOD2) signaling, which results in enhanced Th1 and Th17 responses.[61] These findings are in agreement with our results showing an increased bacterial translocation to the mLN, joint draining lymph nodes, and the spleen. Given that arthritis is driven by Th1 and Th17 cells,[1] it is possible that bacteria found in peripheral lymphoid tissues promote the differentiation of pro-inflammatory T-helper cells in arthritis. As well as translocation of bacteria, increased gut permeability may allow for the systemic dissemination of immunologically active bacterial metabolites or other bacterial products. For instance, children with oligo- and poly-articular juvenile idiopathic arthritis (JIA) show evidence of increased systemic exposure to bacterial products.[29] As a consequence of the increased gut permeability, we detect translocation of LPS, which may itself be driving peripheral inflammation, because LPS is used as an adjuvant to generate inflammatory arthritis in collagen-antibody-induced arthritis in BALB/c mice.[62]

In homeostasis, any effects of locally expressed IFNγ on intestinal permeability should be kept in check by the ability of IL-10 to promote normal barrier function.[16] *In vitro*, IL-10 signaling inhibits IFNγ-mediated disruption of epithelial cell tight junctions and loss of cell viability.[21,43,63] However, IFNγ has been reported to upregulate IL-10R expression by epithelial cells, making them more sensitive to IL-10-mediated protection.[45] In contrast to these *in vitro* findings, in both models of arthritis, we observed a loss of *in vivo* IL-10R expression on the intestinal epithelium and an increase in IFNγ expression by leukocytes. Similarly, using *in vitro* cell cultures of intestinal organoids, we show that exposure of IECs to IFNγ decreases cell survival and IL-10R expression. Adding IL-10 together with IFNγ only partially rescues cell survival. Thus, these results propose for the first time that IFNγ inhibits IL-10R expression by epithelial cells. The discrepancy between our results and those previously shown could be due to the influence of broader changes to the cytokine environment *in vivo* in arthritis and *in vitro* to differences in the responses of cell lines and primary cells cultured as organoids to inflammatory stimuli. The relevance of the loss of epithelial cell IL-10-IL10R gut signaling to the pathogenesis of arthritis was confirmed by our data showing that chimeric mice lacking IL-10R on non-hematopoietic cells developed increased intestinal permeability and inflammation and a significantly more

severe arthritis than WT controls. The loss of intestinal epithelial IL-10R expression seen in arthritis may also be due to dysbiosis, given that, in both models of arthritis, we observed increased abundance of arthritis associated *Prevotella* genus bacteria. This hypothesis is supported by our results showing a recovery of IL-10R expression on intestinal epithelial cells in mice treated with antibiotics during arthritis induction.

Our results show for the first time that a genetic predisposition to gut permeability is pathogenic in a mouse model of arthritis, as claudin-8$^{-/-}$ mice develop a more severe arthritis than WT controls. Further, we demonstrate that preventing the development of intestinal permeability is an effective way of reducing joint swelling in a mouse model of arthritis, confirming results by Tajik et al.[52] in a collagen-induced arthritis model. To prevent the development of gut permeability during arthritis, we treated mice with the zonulin inhibitor AT-1001, also known as larazotide acetate.[49] In the gut, zonulin is expressed by epithelial cells and signals via co-binding protease-activated receptor 2 and epidermal growth factor receptor. This results in protein-kinase-C-α-dependent disruption of tight junction assembly. To this point, the only stimuli reported to elicit zonulin expression by epithelial cells are gluten and bacterial fragments, the latter via C–X–C motif chemokine receptor 3 (CXCR3).[64] Thus, AT-1001 treatment is potentially acting to prevent dysbiosis-induced arthritic pathology. A similar mechanism has been suggested for the appearance of gut permeability in AS, where patients with chronic disease display both bacterial invasion of the ileum and tissue upregulation of zonulin expression.[22] Larazotide acetate is currently in phase 3 trials as a therapy for celiac disease and could be repurposed as an adjunct therapy for RA.[65]

In conclusion, we show that changes in gut morphology and an increase in gut permeability coincide with the initiation of arthritis and abate during the remission from disease. We suggest that changes to the gut are driven by intestinal dysbiosis and loss of tolerance in the periphery and that these changes feedback to enhance systemic inflammation and joint swelling. Finally, modulation of gut permeability or prevention of gut-tropic cellular recirculation directly influences the severity of joint inflammation, thus proposing two novel gut-dependent therapeutic approaches for the treatment of RA.

Limitations of study

There are a few limitations of this study. First of all, although modulating the degree of gut permeability affects the degree of joint swelling, as discussed above, the exact mechanisms by which these two phenomena are linked remain to be fully elucidated. In particular, although we observe increased systemic bacterial translocation in arthritis, we do not know the identity of these bacteria and whether they can directly induce disease pathology. Further, although we moved to the AIA model on a C57BL/6 background to make use of transgenic mice that would allow us to investigate individual elements of gut pathology in arthritis, this model only recapitulates some aspects of human disease. In common with all arthritis models on this background, AIA is an induced model and disease is self-remitting. More work will be needed to determine the effects of alterations to the gut in chronic arthritis and whether gut-directed treatments will be effective in established disease.

STAR★METHODS

Detailed methods are provided in the online version of this paper and include the following:

- KEY RESOURCES TABLE
- RESOURCE AVAILABILITY

- Lead contact
- Materials availability
- Data and code availability
- EXPERIMENTAL MODEL AND SUBJECT DETAILS
  - Human samples
  - Mice
- METHOD DETAILS
  - Induction of antigen-induced arthritis (AIA)
  - *In vivo* intestinal permeability measurements
  - Cell preparation
  - Flow cytometry
  - Histological assessment of frozen tissue sections
  - Periodic Acid-Schiff (PAS) staining
  - Immunofluorescence
  - Bacterial translocation measurement
  - Arthritic cell transfer and bacterial reconstitution
  - Intestinal epithelial cell isolation
  - Gene expression by RT-PCR
  - Small intestine organoid culture
  - Generation of bone marrow chimeras
  - Modulation of intestinal permeability using AT-1001
  - Modulation of immune cell recirculation to the intestine
  - 16S rDNA sequencing
- QUANTIFICATION AND STATISTICAL ANALYSIS

SUPPLEMENTAL INFORMATION

Supplemental information can be found online at https://doi.org/10.1016/j.medj.2021.04.013.

ACKNOWLEDGMENTS

We would like to thank Professors Diane Mathis and Christophe Benoist (Harvard Medical School) for providing the KRN mice, Professor Mauro Peretti (Queen Mary University London) for NOD mice, Professor Werner Muller (University of Manchester) for *Il10ra*$^{-/-}$ mice, the staff of the University College London Biological Services unit for all the help with animal husbandry, and the ABIRISK Consortium for etanercept-treated-patient serum samples. The graphical abstract was created with BioRender.com. This work is funded by a Versus Arthritis program grant (21140) and UKRI/MRC (MR/T000910/1) to C.M. and P.A.B. D.E.M. is funded by a Versus Arthritis PhD studentship (21257) to C.M. E.C.R. is funded by a Versus Arthritis UK Foundation fellowship (21141). L.M. was funded by a Versus Arthritis project grant (21786) to C.M. A.M.S. and B.N.-S. were funded by the Medical Research Council (MR/L000261/1 and MR/K023624/1, respectively).

AUTHOR CONTRIBUTIONS

D.E.M. designed and performed experiments, analyzed data, and wrote the manuscript. M.M. performed experiments, analyzed data, and critically reviewed the manuscript. D.G.A. and N.K. performed 16S rDNA sequencing and analysis. A.M.S. and B.N.-S. provided the *claudin8*$^{-/-}$ mice and critically reviewed the manuscript. A.F. provided AT-1001 and critically reviewed the manuscript. L.M. and J.J.M. recruited RA patients and processed samples. A.D. performed experiments. S.B., A.G., and S.H.-B.-A. provided etanercept samples, and S.B. provided their clinical information. E.C.R. performed experiments and critically reviewed the manuscript. P.A.B. performed experiments, designed the study and experiments, analyzed

data, and wrote the manuscript. C.M. designed the study and experiments, analyzed data, and wrote the manuscript.

DECLARATION OF INTERESTS

A.F. is co-founder and stockholder of Alba Therapeutics, a company developing treatments complementary to the gluten-free diet by exploiting gut permeability.

Received: February 4, 2020
Revised: February 22, 2021
Accepted: April 15, 2021
Published: May 13, 2021

REFERENCES

1. McInnes, I.B., and Schett, G. (2011). The pathogenesis of rheumatoid arthritis. N. Engl. J. Med. *365*, 2205–2219.

2. Horta-Baas, G., Romero-Figueroa, M.D.S., Montiel-Jarquín, A.J., Pizano-Zárate, M.L., García-Mena, J., and Ramírez-Durán, N. (2017). Intestinal dysbiosis and rheumatoid arthritis: a link between gut microbiota and the pathogenesis of rheumatoid arthritis. J. Immunol. Res. *2017*, 4835189.

3. Jeong, Y., Kim, J.W., You, H.J., Park, S.J., Lee, J., Ju, J.H., Park, M.S., Jin, H., Cho, M.L., Kwon, B., et al. (2019). Gut microbial composition and function are altered in patients with early rheumatoid arthritis. J. Clin. Med. *8*, 693.

4. Scher, J.U., Sczesnak, A., Longman, R.S., Segata, N., Ubeda, C., Bielski, C., Rostron, T., Cerundolo, V., Pamer, E.G., Abramson, S.B., et al. (2013). Expansion of intestinal Prevotella copri correlates with enhanced susceptibility to arthritis. eLife *2*, e01202.

5. Drago, L. (2019). *Prevotella copri* and microbiota in rheumatoid arthritis: fully convincing evidence? J. Clin. Med. *8*, 1837.

6. Maeda, Y., Kurakawa, T., Umemoto, E., Motooka, D., Ito, Y., Gotoh, K., Hirota, K., Matsushita, M., Furuta, Y., Narazaki, M., et al. (2016). Dysbiosis contributes to arthritis development via activation of autoreactive T cells in the intestine. Arthritis Rheumatol. *68*, 2646–2661.

7. Maeda, Y., and Takeda, K. (2017). Role of gut microbiota in rheumatoid arthritis. J. Clin. Med. *6*, 60.

8. Pianta, A., Arvikar, S., Strle, K., Drouin, E.E., Wang, Q., Costello, C.E., and Steere, A.C. (2017). Evidence of the immune relevance of Prevotella copri, a gut microbe, in patients with rheumatoid arthritis. Arthritis Rheumatol. *69*, 964–975.

9. Bennike, T.B., Ellingsen, T., Glerup, H., Bonderup, O.K., Carlsen, T.G., Meyer, M.K., Bøgsted, M., Christiansen, G., Birkelund, S., Andersen, V., and Stensballe, A. (2017). Proteome analysis of rheumatoid arthritis gut mucosa. J. Proteome Res. *16*, 346–354.

10. Zhang, X., Zhang, D., Jia, H., Feng, Q., Wang, D., Liang, D., Wu, X., Li, J., Tang, L., Li, Y., et al. (2015). The oral and gut microbiomes are perturbed in rheumatoid arthritis and partly normalized after treatment. Nat. Med. *21*, 895–905.

11. Block, K.E., Zheng, Z., Dent, A.L., Kee, B.L., and Huang, H. (2016). Gut microbiota regulates K/BxN autoimmune arthritis through follicular helper T but not Th17 cells. J. Immunol. *196*, 1550–1557.

12. Guerreiro, C.S., Calado, Â., Sousa, J., and Fonseca, J.E. (2018). Diet, microbiota, and gut permeability-the unknown triad in rheumatoid arthritis. Front. Med. (Lausanne) *5*, 349.

13. Groschwitz, K.R., and Hogan, S.P. (2009). Intestinal barrier function: molecular regulation and disease pathogenesis. J. Allergy Clin. Immunol. *124*, 3–20, quiz 21–22.

14. Capaldo, C.T., Powell, D.N., and Kalman, D. (2017). Layered defense: how mucus and tight junctions seal the intestinal barrier. J. Mol. Med. (Berl.) *95*, 927–934.

15. Corfield, A.P. (2018). The interaction of the gut microbiota with the mucus barrier in health and disease in human. Microorganisms *6*, 78.

16. Andrews, C., McLean, M.H., and Durum, S.K. (2018). Cytokine tuning of intestinal epithelial function. Front. Immunol. *9*, 1270.

17. Bruewer, M., Luegering, A., Kucharzik, T., Parkos, C.A., Madara, J.L., Hopkins, A.M., and Nusrat, A. (2003). Proinflammatory cytokines disrupt epithelial barrier function by apoptosis-independent mechanisms. J. Immunol. *171*, 6164–6172.

18. Chen, J., Wright, K., Davis, J.M., Jeraldo, P., Marietta, E.V., Murray, J., Nelson, H., Matteson, E.L., and Taneja, V. (2016). An expansion of rare lineage intestinal microbes characterizes rheumatoid arthritis. Genome Med. *8*, 43.

19. Bruewer, M., Utech, M., Ivanov, A.I., Hopkins, A.M., Parkos, C.A., and Nusrat, A. (2005). Interferon-gamma induces internalization of epithelial tight junction proteins via a macropinocytosis-like process. FASEB J. *19*, 923–933.

20. Wang, F., Schwarz, B.T., Graham, W.V., Wang, Y., Su, L., Clayburgh, D.R., Abraham, C., and Turner, J.R. (2006). IFN-gamma-induced TNFR2 expression is required for TNF-dependent intestinal epithelial barrier dysfunction. Gastroenterology *131*, 1153–1163.

21. Shouval, D.S., Ouahed, J., Biswas, A., Goettel, J.A., Horwitz, B.H., Klein, C., Muise, A.M., and Snapper, S.B. (2014). Interleukin 10 receptor signaling: master regulator of intestinal mucosal homeostasis in mice and humans. Adv. Immunol. *122*, 177–210.

22. Ciccia, F., Guggino, G., Rizzo, A., Alessandro, R., Luchetti, M.M., Milling, S., Saieva, L., Cypers, H., Stampone, T., Di Benedetto, P., et al. (2017). Dysbiosis and zonulin upregulation alter gut epithelial and vascular barriers in patients with ankylosing spondylitis. Ann. Rheum. Dis. *76*, 1123–1132.

23. Mielants, H., De Vos, M., Goemaere, S., Schelstraete, K., Cuvelier, C., Goethals, K., Maertens, M., Ackerman, C., and Veys, E.M. (1991). Intestinal mucosal permeability in inflammatory rheumatic diseases. II. Role of disease. J. Rheumatol. *18*, 394–400.

24. Smith, M.D., Gibson, R.A., and Brooks, P.M. (1985). Abnormal bowel permeability in ankylosing spondylitis and rheumatoid arthritis. J. Rheumatol. *12*, 299–305.

25. Bosi, E., Molteni, L., Radaelli, M.G., Folini, L., Fermo, I., Bazzigaluppi, E., Piemonti, L., Pastore, M.R., and Paroni, R. (2006). Increased intestinal permeability precedes clinical onset of type 1 diabetes. Diabetologia *49*, 2824–2827.

26. Buscarinu, M.C., Cerasoli, B., Annibali, V., Policano, C., Lionetto, L., Capi, M., Mechelli, R., Romano, S., Fornasiero, A., Mattei, G., et al. (2017). Altered intestinal permeability in patients with relapsing-remitting multiple sclerosis: A pilot study. Mult. Scler. *23*, 442–446.

27. Camara-Lemarroy, C.R., Silva, C., Greenfield, J., Liu, W.Q., Metz, L.M., and Yong, V.W. (2020). Biomarkers of intestinal barrier function in multiple sclerosis are associated with disease activity. Mult. Scler. *26*, 1340–1350.

28. Dhillon, A.K., Kummen, M., Trøseid, M., Åkra, S., Liaskou, E., Moum, B., Vesterhus, M., Karlsen, T.H., Seljeflot, I., and Hov, J.R. (2019). Circulating markers of gut barrier function associated with disease severity in primary sclerosing cholangitis. Liver Int. *39*, 371–381.

29. Fotis, L., Shaikh, N., Baszis, K.W., Samson, C.M., Lev-Tzion, R., French, A.R., and Tarr, P.I. (2017). Serologic evidence of gut-driven systemic inflammation in juvenile idiopathic arthritis. J. Rheumatol. *44*, 1624–1631.

30. Pelsers, M.M., Hermens, W.T., and Glatz, J.F. (2005). Fatty acid-binding proteins as plasma

markers of tissue injury. Clin. Chim. Acta 352, 15–35.

31. Mielants, H., Goemaere, S., De Vos, M., Schelstraete, K., Goethals, K., Maertens, M., Ackerman, C., and Veys, E.M. (1991). Intestinal mucosal permeability in inflammatory rheumatic diseases. I. Role of antiinflammatory drugs. J. Rheumatol. 18, 389–393.

32. Iwata, M., Hirakiyama, A., Eshima, Y., Kagechika, H., Kato, C., and Song, S.Y. (2004). Retinoic acid imprints gut-homing specificity on T cells. Immunity 21, 527–538.

33. Wurbel, M.A., Malissen, M., Guy-Grand, D., Meffre, E., Nussenzweig, M.C., Richelme, M., Carrier, A., and Malissen, B. (2001). Mice lacking the CCR9 CC-chemokine receptor show a mild impairment of early T- and B-cell development and a reduction in T-cell receptor gammadelta(+) gut intraepithelial lymphocytes. Blood 98, 2626–2632.

34. Kouskoff, V., Korganow, A.S., Duchatelle, V., Degott, C., Benoist, C., and Mathis, D. (1996). Organ-specific disease provoked by systemic autoimmunity. Cell 87, 811–822.

35. Stevenson, B.R., Siliciano, J.D., Mooseker, M.S., and Goodenough, D.A. (1986). Identification of ZO-1: a high molecular weight polypeptide associated with the tight junction (zonula occludens) in a variety of epithelia. J. Cell Biol. 103, 755–766.

36. Wells, J.M., Brummer, R.J., Derrien, M., MacDonald, T.T., Troost, F., Cani, P.D., Theodorou, V., Dekker, J., Méheust, A., de Vos, W.M., et al. (2017). Homeostasis of the gut barrier and potential biomarkers. Am. J. Physiol. Gastrointest. Liver Physiol. 312, G171–G193.

37. Erben, U., Loddenkemper, C., Doerfel, K., Spieckermann, S., Haller, D., Heimesaat, M.M., Zeitz, M., Siegmund, B., and Kühl, A.A. (2014). A guide to histomorphological evaluation of intestinal inflammation in mouse models. Int. J. Clin. Exp. Pathol. 7, 4557–4576.

38. Holmén Larsson, J.M., Thomsson, K.A., Rodríguez-Piñeiro, A.M., Karlsson, H., and Hansson, G.C. (2013). Studies of mucus in mouse stomach, small intestine, and colon. III. Gastrointestinal Muc5ac and Muc2 mucin O-glycan patterns reveal a regiospecific distribution. Am. J. Physiol. Gastrointest. Liver Physiol. 305, G357–G363.

39. Wu, H.J., Ivanov, I.I., Darce, J., Hattori, K., Shima, T., Umesaki, Y., Littman, D.R., Benoist, C., and Mathis, D. (2010). Gut-residing segmented filamentous bacteria drive autoimmune arthritis via T helper 17 cells. Immunity 32, 815–827.

40. Rakoff-Nahoum, S., Paglino, J., Eslami-Varzaneh, F., Edberg, S., and Medzhitov, R. (2004). Recognition of commensal microflora by toll-like receptors is required for intestinal homeostasis. Cell 118, 229–241.

41. Oleinika, K., Rosser, E.C., Matei, D.E., Nistala, K., Bosma, A., Drozdov, I., and Mauri, C. (2018). CD1d-dependent immune suppression mediated by regulatory B cells through modulations of iNKT cells. Nat. Commun. 9, 684.

42. Rosser, E.C., Oleinika, K., Tonon, S., Doyle, R., Bosma, A., Carter, N.A., Harris, K.A., Jones, S.A., Klein, N., and Mauri, C. (2014). Regulatory B cells are induced by gut microbiota-driven interleukin-1β and interleukin-6 production. Nat. Med. 20, 1334–1339.

43. Denning, T.L., Campbell, N.A., Song, F., Garofalo, R.P., Klimpel, G.R., Reyes, V.E., and Ernst, P.B. (2000). Expression of IL-10 receptors on epithelial cells from the murine small and large intestine. Int. Immunol. 12, 133–139.

44. Jarry, A., Bossard, C., Bou-Hanna, C., Masson, D., Espaze, E., Denis, M.G., and Laboisse, C.L. (2008). Mucosal IL-10 and TGF-beta play crucial roles in preventing LPS-driven, IFN-gamma-mediated epithelial damage in human colon explants. J. Clin. Invest. 118, 1132–1142.

45. Kominsky, D.J., Campbell, E.L., Ehrentraut, S.F., Wilson, K.E., Kelly, C.J., Glover, L.E., Collins, C.B., Bayless, A.J., Saeedi, B., Dobrinskikh, E., et al. (2014). IFN-γ-mediated induction of an apical IL-10 receptor on polarized intestinal epithelia. J. Immunol. 192, 1267–1276.

46. Angelow, S., and Yu, A.S. (2009). Structure-function studies of claudin extracellular domains by cysteine-scanning mutagenesis. J. Biol. Chem. 284, 29205–29217.

47. Zeissig, S., Bürgel, N., Günzel, D., Richter, J., Mankertz, J., Wahnschaffe, U., Kroesen, A.J., Zeitz, M., Fromm, M., and Schulzke, J.D. (2007). Changes in expression and distribution of claudin 2, 5 and 8 lead to discontinuous tight junctions and barrier dysfunction in active Crohn's disease. Gut 56, 61–72.

48. Fasano, A. (2012). Zonulin, regulation of tight junctions, and autoimmune diseases. Ann. N Y Acad. Sci. 1258, 25–33.

49. Leffler, D.A., Kelly, C.P., Green, P.H., Fedorak, R.N., DiMarino, A., Perrow, W., Rasmussen, H., Wang, C., Bercik, P., Bachir, N.M., and Murray, J.A. (2015). Larazotide acetate for persistent symptoms of celiac disease despite a gluten-free diet: a randomized controlled trial. Gastroenterology 148, 1311, 9.e6.

50. Bekker, P., Ebsworth, K., Walters, M.J., Berahovich, R.D., Ertl, L.S., Charvat, T.T., Punna, S., Powers, J.P., Campbell, J.J., Sullivan, T.J., et al. (2015). CCR9 antagonists in the treatment of ulcerative colitis. Mediators Inflamm. 2015, 628340.

51. Bjarnason, I., Williams, P., So, A., Zanelli, G.D., Levi, A.J., Gumpel, J.M., Peters, T.J., and Ansell, B. (1984). Intestinal permeability and inflammation in rheumatoid arthritis: effects of non-steroidal anti-inflammatory drugs. Lancet 2, 1171–1174.

52. Tajik, N., Frech, M., Schulz, O., Schälter, F., Lucas, S., Azizov, V., Dürholz, K., Steffen, F., Omata, Y., Rings, A., et al. (2020). Targeting zonulin and intestinal epithelial barrier function to prevent onset of arthritis. Nat. Commun. 11, 1995.

53. Picchianti-Diamanti, A., Panebianco, C., Salemi, S., Sorgi, M.L., Di Rosa, R., Tropea, A., Sgrulletti, M., Salerno, G., Terracciano, F., D'Amelio, R., et al. (2018). Analysis of gut microbiota in rheumatoid arthritis patients: disease-related dysbiosis and modifications induced by etanercept. Int. J. Mol. Sci. 19, 2938.

54. Hegan, P.S., Chandhoke, S.K., Barone, C., Egan, M., Bähler, M., and Mooseker, M.S. (2016). Mice lacking myosin IXb, an inflammatory bowel disease susceptibility gene, have impaired intestinal barrier function and superficial ulceration in the ileum. Cytoskeleton (Hoboken) 73, 163–179.

55. Papp, M., Foldi, I., Nemes, E., Udvardy, M., Harsfalvi, J., Altorjay, I., Mate, I., Dinya, T., Varvolgyi, C., Barta, Z., et al. (2008). Haptoglobin polymorphism: a novel genetic risk factor for celiac disease development and its clinical manifestations. Clin. Chem. 54, 697–704.

56. Adegbola, S.O., Sahnan, K., Warusavitarne, J., Hart, A., and Tozer, P. (2018). Anti-TNF therapy in Crohn's disease. Int. J. Mol. Sci. 19, 2244.

57. Ma, X., and Xu, S. (2013). TNF inhibitor therapy for rheumatoid arthritis. Biomed. Rep. 1, 177–184.

58. Segal, A.W., Isenberg, D.A., Hajirousou, V., Tolfree, S., Clark, J., and Snaith, M.L. (1986). Preliminary evidence for gut involvement in the pathogenesis of rheumatoid arthritis? Br. J. Rheumatol. 25, 162–166.

59. Porzio, V., Biasi, G., Corrado, A., De Santi, M., Vindigni, C., Viti, S., Bayeli, P.F., and Marcolongo, R. (1997). Intestinal histological and ultrastructural inflammatory changes in spondyloarthropathy and rheumatoid arthritis. Scand. J. Rheumatol. 26, 92–98.

60. Flak, M.B., Colas, R.A., Muñoz-Atienza, E., Curtis, M.A., Dalli, J., and Pitzalis, C. (2019). Inflammatory arthritis disrupts gut resolution mechanisms, promoting barrier breakdown by Porphyromonas gingivalis. JCI Insight 4, e125191.

61. Costa, F.R., Françozo, M.C., de Oliveira, G.G., Ignacio, A., Castoldi, A., Zamboni, D.S., Ramos, S.G., Câmara, N.O., de Zoete, M.R., Palm, N.W., et al. (2016). Gut microbiota translocation to the pancreatic lymph nodes triggers NOD2 activation and contributes to T1D onset. J. Exp. Med. 213, 1223–1239.

62. Irrera, N., D'Ascola, A., Pallio, G., Bitto, A., Mazzon, E., Mannino, F., Squadrito, V., Arcoraci, V., Minutoli, L., Campo, G.M., et al. (2019). β-caryophyllene mitigates collagen antibody induced arthritis (CAIA) in mice through a cross-talk between CB2 and PPAR-γ receptors. Biomolecules 9, 326.

63. Alexeev, E.E., Lanis, J.M., Kao, D.J., Campbell, E.L., Kelly, C.J., Battista, K.D., Gerich, M.E., Jenkins, B.R., Walk, S.T., Kominsky, D.J., and Colgan, S.P. (2018). Microbiota-derived indole metabolites promote human and murine intestinal homeostasis through regulation of interleukin-10 receptor. Am. J. Pathol. 188, 1183–1194.

64. Fasano, A. (2011). Zonulin and its regulation of intestinal barrier function: the biological door to inflammation, autoimmunity, and cancer. Physiol. Rev. 91, 151–175.

65. 9 Meters Biopharma (2021). Study to evaluate the efficacy and safety of larazotide acetate for the relief of CeD symptoms. https://ClinicalTrials.gov/show/NCT03569007.

66. Schloss, P.D., Westcott, S.L., Ryabin, T., Hall, J.R., Hartmann, M., Hollister, E.B., Lesniewski, R.A., Oakley, B.B., Parks, D.H.,

CellPress
OPEN ACCESS

Robinson, C.J., et al. (2009). Introducing mothur: open-source, platform-independent, community-supported software for describing and comparing microbial communities. Appl. Environ. Microbiol. *75*, 7537–7541.

67. Krausgruber, T., Schiering, C., Adelmann, K., Harrison, O.J., Chomka, A., Pearson, C., Ahern, P.P., Shale, M., Oukka, M., and Powrie, F. (2016). T-bet is a key modulator of IL-23-driven pathogenic CD4(+) T cell responses in the intestine. Nat. Commun. *7*, 11627.

68. Jowett, G.M., Norman, M.D.A., Yu, T.T.L., Rosell Arévalo, P., Hoogland, D., Lust, S.T., Read, E., Hamrud, E., Walters, N.J., Niazi, U., et al. (2021). ILC1 drive intestinal epithelial and matrix remodelling. Nat. Mater. *20*, 250–259.

69. Zietek, T., and Rath, E. (2018). Chapter 3 - Intestinal organoids: mini-guts grown in the laboratory. In Organoids and Mini-organs, J.A. Davies and M.L. Lawrence, eds. (Academic), pp. 43–71.

70. Rosser, E.C., Piper, C.J.M., Matei, D.E., Blair, P.A., Rendeiro, A.F., Orford, M., Alber, D.G., Krausgruber, T., Catalan, D., Klein, N., et al. (2020). Microbiota-derived metabolites suppress arthritis by amplifying aryl-hydrocarbon receptor activation in regulatory B cells. Cell Metab. *31*, 837–851.e10.

STAR★METHODS

KEY RESOURCES TABLE

REAGENT or RESOURCE	SOURCE	IDENTIFIER
Antibodies		
CD19 BV785, Clone 6D5	Biolegend	Cat# 115543; RRID: AB_11218994
CD3 BV605, Clone 17A2	Biolegend	Cat# 100237; RRID: AB_2562039
CD4 BV711, Clone RM4-5	Biolegend	Cat# 100550; RRID: AB_2562099
CD8a BUV805, Clone 53-6.7	BD Biosciences	Cat#; 564920 RRID: AB_2716856
MHC-II APC-Cy7, Clone M5/114.15.2	Biolegend	Cat# 107628 RRID: AB_2069377
Ly6G Alexa Fluor 700, Clone 1A8	Biolegend	Cat# 127622 RRID: AB_10643269
EpCAM FITC, Clone G8.8	Biolegend	Cat# 118208 RRID: AB_1134107
CCR9 PE-Cy7, Clone CW-1.2	Biolegend	Cat# 128712 RRID: AB_10933082
CD11b BV605, Clone M1/70	Biolegend	Cat# 101257; RRID: AB_2565431
CD11c FITC, Clone N418	Biolegend	Cat# 117306; RRID: AB_313775
CD45 BUV737, Clone 104	BD Biosciences	Cat# 564880 RRID: AB_2738998
LPAM-1 PE, Clone DATK32	BD Biosciences	Cat# 553811 RRID: AB_395066
IL-10R PE, Clone 1B1.3a	BD Biosciences	Cat# 112706 RRID: AB_313519
Ly6C PE-Cy7, Clone HK1.4	ThermoFisher Scientific	Cat# 25-5932-82 RRID: AB_2573503
F4/80 APC, Clone BM8	ThermoFisher Scientific	Cat# 17-4801-82: RRID: AB_2784648
IFNγ APC, Clone XMG1.2	BD Biosciences	Cat# 17-7311-82: RRID: AB_469504
IL-10 PE, Clone JES5-16E3	Biolegend	Cat# 505008; RRID: AB_315362
Il-17 eFluor 450, Clone eBio17B7	ThermoFisher Scientific	Cat# 48-7177-82 RRID: AB_11149503
CCR9 Alexa Fluor 647, Clone BLICCR9	Biolegend	Cat# 346301 RRID: AB_2275427
Rabbit anti-mouse ZO-1	Abcam	Cat# ab216880; RRID: AB_10678863
Rabbit polyclonal anti-MUC2	Abcam	Cat# ab76774 RRID: AB_1523987
Goat anti-rabbit IgG Alexa Fluore 555	ThermoFisher Scientific	Cat# A32732 RRID: AB_2633281
Donkey anti-rabbit IgG Alexa Fluor 488	ThermoFisher Scientific	Cat# A32790 RRID: AB_2762833
Chemicals, peptides, and recombinant proteins		
Methylated bovine serum albumin (mBSA)	Sigma Aldrich	Cat# A1009
Immunisation Grade Bovine Type II Collagen	Chondrex	Cat# 20021
Incomplete Freund's adjuvant (IFA)	Sigma Aldrich	Cat# F5506
Phorbol-12-myristate-13 acetate (PMA)	Sigma Aldrich	Cat# P8139
Ionomycin	Sigma Aldrich	Cat# I0634
DAPI	Sigma Aldrich	Cat# D9542
Brefeldin A	Biolegend	Cat# 420601
2-Mercaptoethanol	ThermoFisher Scientific	Cat# 31350010
RNase-Free DNase set	QIAGEN	Cat# 79254
Neomycin	Sigma Aldrich	Cat# N1876
Metronidazole	Sigma Aldrich	Cat# M3761
Vancomycin	Sigma Aldrich	Cat# V2002
Ampicillin	Sigma Aldrich	Cat# A9393
FITC-dextran	Sigma Aldrich	Cat# 46944-500MG-F
Collagenase VIII	Sigma Aldrich	Cat# C2139
Collagenase V	Sigma Aldrich	Cat# C9263
Collagenase D	Roche Diagnostics	Cat# 11088866001
Dispase	GIBCO	Cat# 17105-041
DNase I	Roche Diagnostics	Cat# 10104159001
Liberase TL	Roche Diagnostics	Cat# 5401020001
GlutaMAX	ThermoFisher Scientific	Cat# 35050061
N-2	ThermoFisher Scientific	Cat# 17502048
B27	ThermoFisher Scientific	Cat# 17504044
N-acetylcysteine	Sigma Aldrich	Cat# A9165
Murine epithelial growth factor	Peprotech	Cat# 315-09
Human recombinant R-spondin	Biolegend	Cat# 783604

(*Continued on next page*)

Continued

REAGENT or RESOURCE	SOURCE	IDENTIFIER
Murine recombinant Noggin	Peprotech	Cat# 250-38
Recombinant murine IFNγ	Biolegend	Cat# 575306
Recombinant murine IL-10	Biolegend	Cat# 575806
Vercirnon (anti-CCR9)	Chemocentryx/MedChemExpress	Cat# HY-15724
Critical commercial assays		
Picopure™ RNA isolation kit	ThermoFisher Scientific	Cat# KIT0204
iScript™ cDNA synthesis kit	Biorad	Cat# 1708891
iQ™ SYBR® green supermix	Biorad	Cat# 1708882
Nextera DNA library preparation kit	Illumina	Cat# FC-121-1030
MinElute PCR purification kit	QIAGEN	Cat# 28004
QIAamp DNA Mini Kit	QIAGEN	Cat# 51304
BioPulverizer Lysing Matrix E	MP Biomedical Europe	Cat# 116914050
Taq PCR Core kit	QIAGEN	Cat# 201225
ZymoBIOMICS Microbial Community DNA Standard	Zymo Research	Cat# D6305
Agencourt AMPure XP	Beckman Coulter	Cat# A63881
Qubit dsDNA HS Assay Kit-500 assays	ThermoFisher Scientific	Cat# Q32854
NEBNext Library Quant Kit for Illumina	New England BioLabs	Cat# E7630L
High Sensitivity D1000 ScreenTape	Agilent	Cat# 5067-5584
Agilent High Sensitivity DNA Reagents	Agilent	Cat# 5067-4627
MiSeq Reagent Kit v2 (500-cycles)	Illumina	Cat# MS-102-2003
PhiX Control v3	Illumina	Cat# FC-110-3001
Human LPS-binding protein ELISA kit	R&D Systems	Cat# DY870-05
Human intestinal fatty acid binding protein (I-FABP) ELISA kit	Sigma Aldrich	Cat# RAB0537
Human LPS ELISA kit	Cusabio	Cat# CSB-E09945h
Murine LPS-binding protein ELISA kit	Hycult Biotech	Cat# HK205-01
PAS staining kit	Abcam	Ab150680
Deposited data		
16S DATA	This paper	NCBI SRA accession number PRJNA720523
Experimental models: Organisms/strains		
Mouse, C57BL/6J	Envigo	N/A
Mouse, NOD.Cg-*Prkdc^{scid}Il2rg^{tm1Wjl}/Szj* (NSG)	UCL, Clare Hall	N/A
Mouse, *Tg(TcraR28,TcrbR28)KRNDim*	Prof. Diane Mathis	N/A
Mouse, NOD I-A^{g7}	Prof. Mauro Peretti	N/A
Mouse, *Il10ra$^{-/-}$*	Prof. Werner Muller	N/A
Mouse, *B6;129S5-Cldn8^{tm1Lex}/Mmucd*	Prof. Andrew Smith	N/A
Oligonucleotides		
qPCR primers		
Hprt		
Fwd 5′– TTTGCTGACCTGCTGGATTAC –3′	ThermoFisher Scientific, This paper	N/A
Rev 5′- CTTTTATGTCCCCCGTTGACT –3′		
V3/V4 16 s		
Fwd 5′–TCCTACGGGAGGCAGCAGT-3′	ThermoFisher Scientific, This paper	N/A
Rev 5′-GGACTACCAGGGTATCTAATCCTGTT-3′		
Il10ra		
N/A	QIAGEN	Cat# 249900
Software and algorithms		
GraphPad Prism 8	Graphpad Software	https://www.graphpad.com
Flowjo v10.5.0	Flowjo, LLC	https://www.flowjo.com
NDP.view2 Viewing software	Hamamatsu	https://www.hamamatsu.com/eu/en/product/type/U12388-01/index.html
Illumina Casava 1.7	Illumina	https://www.illumina.com
Mothur V1.35.13	Schloss et al.[66]	https://mothur.org/
Phyloseq		https://joey711.github.io/phyloseq/

(*Continued on next page*)

Continued

REAGENT or RESOURCE	SOURCE	IDENTIFIER
Other		
RPMI-1640 media	Sigma Aldrich	Cat# R8758
Advanced DMEM/F12	GIBCO	Cat# 12634028
Red blood cell lysis buffer	Sigma Aldrich	Cat# R7757
Foetal calf serum (FCS)	Biosera	Cat# FB1001/500
Normal Goat Serum	Vector	Cat# S1000
Ethylenediaminetetraacetic acid	ThermoFisher Scientific	Cat# AM9260G
LIVE/DEAD™ Fixable Blue	Invivogen	Cat# L34961
Vectashield Mounting Medium with DAPI	Vector labs	Cat# H-1200-10
Formalin solution, neutral buffered, 10%	Sigma Aldrich	Cat# HT501320
Penicillin/Streptomycin	Sigma Aldrich	Cat# P0781
eBioscience™ Intracellular fixation & permeabilisation buffer set	ThermoFisher Scientific	Cat# P078188-8824-00
Brilliant stain buffer	BD Biosciences	Cat# 563794
M. tuberculosis H37 Ra, desiccated	BD	Cat# 231141
Percoll Plus	GE Healthcare	Cat# 17544501
O.C.T compound	Tissue-Tek	Cat# 23-730-571
Matrigel	Corning	Cat# 356231

RESOURCE AVAILABILITY

Lead contact

Further information and requests for resources and reagents should be directed to and will be fulfilled by the lead contact, Paul Blair (p.blair@ucl.ac.uk).

Materials availability

This study did not generate new unique reagents.

Data and code availability

The 16S dataset generated during this study is available at NCBI Sequence Read Archive: PRJNA720523.

EXPERIMENTAL MODEL AND SUBJECT DETAILS

Human samples

Serum was obtained from rheumatoid arthritis (RA) patients and healthy adult controls with fully informed consent as approved by the ethics committee of: University College London Hospitals Health Service Trust (14/LO/0506 and 14/SC/1200); CPP, Ile de France VII (13-048), Academic Medical Centre, Amsterdam (METC 2013_304), and Azienda Ospedaliero Universitaria Careggi (2012/0035P82). Clinical information for patients and healthy controls is shown in Table S1.

Mice

C57BL/6J mice were purchased from Envigo Laboratories. NOD *scid* gamma (NSG, NOD.Cg-*Prkdc*^{scid}*Il2rg*^{tm1Wjl}*/Szj*) mice were purchased from UCL, Clare Hall. K/BxN spontaneous arthritis mice were bred in-house by crossing TCR transgenic KRN mice (*Tg(TcraR28,TcrbR28)KRNDim*, provided by Diane Mathis, Harvard Medical School, USA) with (I-A^{g7}) NOD mice (provided by Mauro Peretti, Queen Mary University of London, UK). *Il10ra*$^{-/-}$ mice were provided by W. Muller (University of Manchester, UK). *Claudin8*$^{-/-}$ mice (*B6;129S5-Cldn8*^{tm1Lex}*/Mmucd*) were provided by A. Smith and B. Nedjat-Shokouhi (for genetic information on the strain, Supplemental Methods Figure 1). Mice were sex-matched and mixed sexes were used for all K/BxN experiments, while only females were used for AIA experiments due to predictable incidence of disease. Mice were assigned to experimental groups at

random and, where possible, mixed among cages. Mice were housed in individually ventilated cages at 20-24°C, 45%–64% humidity, and at a 12- light/dark cycle in specific opportunist- and pathogen-free (SOPF) conditions (health screening (Full-FELASA profile) was performed annually). Experimental mice were fed Harlan Teklad pellets 2018 (18% protein) and breeding mice were fed Harlan Teklad pellets 2010 (19% protein) with food and water available *ad libitum*. Mice were used between 2-12 weeks of age and were maintained at animal facilities at University College London. Where possible, initial preliminary experiments were undertaken to establish sample sizes, considering an ethical and reductionist animal use. All experiments were approved by the Animal Welfare and Ethical Review Body of University College London and authorized by the United Kingdom Home Office.

For K/BxN, joint (front paw, rear ankle) size was measured using calipers (POCO 2T; Kroeplin Längenmesstechnik) and their disease scores were calculated as follows:

$$\%\ \text{Swelling} = \frac{\textit{joint size at Tx} - \textit{joint size at T0}*}{\textit{joint size at T0}} \times 100$$

$*T_0$ = 3 weeks of age, day of weaning.

METHOD DETAILS

Induction of antigen-induced arthritis (AIA)

AIA was induced as previously described[41,42]. Briefly, mice were immunized subcutaneously at the base of the tail with 200 μg methylated bovine serum albumin (mBSA, Sigma-Aldrich) in 100 μL complete Freund's adjuvant (CFA) and phosphate-buffered saline (PBS, Sigma-Aldrich). 3 mg/ml CFA was prepared by mixing *Mycobacterium tuberculosis* (231141, BD) in incomplete Freund's adjuvant (F5506, Sigma-Aldrich). After 7 days, mice received an intra-articular injection of 200 μg mBSA in 10 μL PBS in the right knee or PBS alone as a control in the left knee. Joint size was measured using calipers. Knee swelling was calculated as percentage increase in size between antigen-injected knee and PBS-injected knee.

Disease scores were calculated as follows:

$$\%\ \text{Swelling} = \frac{\textit{mBSA knee} - \textit{PBS knee}}{\textit{PBS knee}} \times 100$$

If treated with antibiotics for microbiota depletion during arthritis, C57BL/6 mice were given, in their drinking water, a combination of neomycin (1 g/L, N1876, Sigma-Aldrich), metronidazole (1 g/L, M3761, Sigma-Aldrich), and vancomycin (0.5 g/L, V2002, Sigma-Aldrich), from 7 days prior to the subcutaneous injection. The mice were kept on antibiotics throughout the experiment.

In vivo intestinal permeability measurements

Human LPS-binding protein (LBP) levels were measured by ELISA following the manufacturer's instructions (DY870-05, RnD Systems) in the sera of RA patients and healthy controls (HC) (diluted 1 in 1000 in PBS/1% BSA). The level of intestinal fatty acid binding protein (I-FABP) was also measured in the sera of the mentioned RA patients and HC (1:1) by ELISA according to manufacturer's instructions (RAB0537, Sigma). LPS levels were also measured by ELISA in these samples, undiluted, according to manufacturer's instructions (CSB-E09945h, Cusabio).

Murine LPS-binding protein levels were measured in mouse serum (diluted 1 in 1000) by ELISA following the manufacturer's instructions (HK205-01, Hycult Biotech).

For FITC-dextran translocation measurements, food was removed from cages for 14 h and mice were then gavaged with 600 mg/kg body weight of 4 kD FITC-dextran diluted in water (46944-500MG-F, Sigma). Blood was collected 30 min after gavage and the serum concentration of the FITC-dextran was determined using a fluorometer (TECAN Genios Spectra Fluor plus) with an excitation wavelength of 485 nm and an emission wavelength of 535 nm. FITC-dextran was serially diluted in PBS to establish a standard curve.

Cell preparation

Spleens, Peyer's patches (PP), mesenteric (mLN) and axillary (ALN) lymph nodes were collected post-mortem in complete RPMI-1640 (cRPMI) media with L-glutamine and $NaHCO_3$ (Sigma-Aldrich), with 1:10 fetal bovine serum (FBS) (Biosera) and 1:100 Penicillin (10.000 units/ml) /Streptomycin (10 mg/ml) (Sigma-Aldrich). Single cell suspensions were obtained by dissociating tissues through 70 µm cell strainers (BD Biosciences). Erythrocytes were lysed using Red Blood Cell (RBC) Lysis buffer (Sigma-Aldrich).

For the isolation of intra-epithelial lymphocytes (IEL) and lamina propria mononuclear cells (LPMC), the small and large intestines were removed and cleaned of fat and feces. For small intestines, the PPs were removed and they were divided into duodenum, jejunum and ileum. Intestines were sliced longitudinally and cut into 5-10 mm pieces and incubated with Hanks Balanced Salt Solution (HBSS, Thermo Fisher Scientific) containing 5 mM ethylenediaminetetraacetic acid (EDTA, Thermo Fisher Scientific) for 15 min at 180 rpm and 37°C. After incubation, the samples were vortexed and IELs were collected from the solution through a 100 µm cell strainer (BD Biosciences) and the tissue was re-incubated as above. After both sets of IELs isolation the remaining tissue was washed in fresh HBSS to remove the EDTA, and further incubated with the respective digestion enzymes (small intestine: collagenase VIII – 1 mg/mL (C2139, Sigma-Aldrich); colon: collagenase V - 0.425 mg/mL (C9263, Sigma-Aldrich), collagenase D – 0.625 mg/mL (11088866001, Roche Diagnostics), dispase – 1 mg/mL (17105-041, GIBCO), DNase I – 0.03 mg/mL (10104159001, Roche Diagnostics)). The small intestines were incubated for 25 min and the colons for 40 min at 180 rpm and 37°C, with rigorous shaking every 10 min. The collected IELs were spun through a 40:75% isotonic Percoll Plus (17544501, GE Healthcare) gradient (20 min, 21°C, 900 g) and the interface was further used. For the LPMCs, the filtered and collected cells post-digestion were washed in cRPMI and resuspended in 40% isotonic Percoll and centrifuged at 450 g for 8 min in order to remove debris from samples.

For the isolation of lung immune cells, the lung vascular bed was perfused with PBS prior to dissection. The lungs were cut into small pieces and digested in serum-free RPMI-1640 containing 0.13 mg/ml Liberase TL (Roche) and 20 U/ml DNase I (Roche) for 45 min at 37°C with constant shaking. The digested tissue was further dissociated into single cells through 70 µm cell strainers. The gating strategy for identifying lung immune cells by flow cytometry is given in Supplemental Methods Figure 2.

When all single cell suspensions were collected, they were washed in cRPMI and counted.

Flow cytometry

Single-cell suspensions were stained with combinations of the following surface anti-mouse markers: CD19 BV785, CD3 BV605, CD4 BV711, MHC-II APC-Cy7, Ly6G Alexa Fluor 700, CD11b BV605, CD11c FITC, EpCAM FITC, CCR9 PE-Cy7 (Biolegend), CD45 BUV737, CD8 BUV805, LPAM-1 PE, IL-10R PE, Siglec F PE (BD

Bioscience), Ly6C PE-Cy7 (eBioscience), F4/80 APC (Invitrogen); and LIVE/DEAD Fixable Blue Dead Cell Stain (Invitrogen, Thermo Fisher Scientific) for dead cell exclusion at RT for 30 min and fixed in 2% paraformaldehyde (PFA) solution (Sigma). For intracellular cytokine production, cells were first cultured at 0.5X10^6 cells/well in cRPMI with phorbol 12-myristate 13-acetate (PMA, 50 ng/ml, Sigma-Aldrich), Ionomycin (250 ng/ml, Sigma-Aldrich) and Brefeldin A (5 μg/ml, Sigma-Aldrich) for 4.5 h. Cells were then stained for surface markers as above, then fixed and permeabilized with the eBioscience intracellular staining kit as per manufacturer's instructions, then incubated with combinations of IFNγ APC (BD Bioscience), IL-10 PE (Biolegend), IL-17 eFluor 450 (eBioscience).

PBMCs isolated from healthy controls or RA patients were stained *ex vivo* with LIVE/DEAD Fixable Blue Dead Cell Stain (Invitrogen), for 20 min at RT and anti-human antibodies for flow cytometry, for 30 min at 4°C as follows: CCR9 Alexa Fluor 647 (Biolegend) and LPAM-1 PE (Novus Biologicals).

Flow cytometry was performed on LSR II (BD Biosciences). Data were analyzed using FlowJo (Treestar).

Histological assessment of frozen tissue sections

Intestinal sections (duodenum, jejunum and ileum from the small intestine and proximal, medial and distal colons) were harvested and embedded in O.C.T compound (Tissue-Tek), and lungs were inflated with 50% OCT/PBS and embedded in OCT, and snap-frozen in dry-ice-cold isopentane (Sigma). Intestinal tissues were cut into 6 μm sections (HM525 NX cryostat, Thermo Scientific) and lungs were cut into 10 μm sections and adhered to Superfrost Plus slides (VWR) for hematoxylin and eosin (H&E) staining by the Tissue-Tek DSR automatic stainer. Briefly, the sections were fixed in 10% neutral buffered formalin solution (Sigma), rehydrated in PBS, stained with hematoxylin, washed, counterstained with eosin Y, then washed and dehydrated in sequentially higher concentrations of ethanol from 75% to 100%. The sections were scanned using the NDP NanoZoomer (Hamamatsu) and analyzed with the NDP view software.

The level of intestinal inflammation was semiquantitatively scored in a blinded fashion for each part of the small and large intestines as previously described with minor alterations[37,67]. Briefly, five criteria were graded for each tissue from 0 to 3: leucocyte infiltration (0- none; 1- minimal, 2- slight, multifocal infiltration of lymphocytes and neutrophils in the mucosal layer; 3- moderate, diffuse infiltration of lymphocytes and neutrophils in the mucosal and submucosal layers), crypt elongation, muscle wall thickening (both 0- normal (≤100% of naive or 2-week old K/BxN); 1- slight (101%–150%); 2- moderate (151%–200%); 3- severe (> 200%)), degree of epithelial erosion and loss of goblet cells or entire crypts (0-, none; 1- slight, multifocal; 2- moderate, multifocal; 3- severe, diffuse) in comparison to intestinal sections of naive mice. The scores for each criterion were summed up to give the overall inflammation score of each tissue.

Periodic Acid-Schiff (PAS) staining

Intestinal sections were harvested and snap-frozen in OCT as above and stored at −80°C. The cryo-sections (6 μm) were fixed with 10% neutral buffered formalin solution (Sigma) for 10 min (RT) and stained for mucin and goblet cell counts using a PAS staining kit according to the manufacturer's instructions (ab150680, abcam). Briefly, the sections were stained with periodic acid solution, washed in RO water, stained in Schiff's solution, rinsed under tap water, stained with hematoxylin, bluing reagent

and light green reagent with rinses between each step and finally dehydrated in sequentially higher concentrations of ethanol from 75% to 100% and mounted using DPX (BDH VWR). The sections were scanned using the NDP NanoZoomer (Hamamatsu) and analyzed with the NDP view software.

Immunofluorescence

Intestinal sections and lungs were harvested and snap-frozen in OCT as above. The cryo-sections (6 μm) were fixed with 10% neutral buffered formalin solution (Sigma) for 10 min at room temperature (RT), rehydrated in PBS, then blocked with 10% normal goat (Vector Labs) or donkey (Abcam) serum with 0.3% Triton X-100 (Sigma) for 20 min at room temperature (RT). The tissues were incubated with primary antibodies (EpCAM-FITC, Biolegend; rabbit anti-mouse ZO-1, Abcam; rabbit polyclonal anti-MUC2, Abcam) overnight at 4°C. The secondary antibodies (goat anti-rabbit IgG-Alexa Fluor 555, Thermo Fisher, donkey anti-rabbit IgG-Alexa Fluor 488, Thermo Fisher) were added the following day after X2 PBS washes and incubated for 1 hour at RT. The slides were finally mounted in Prolong Antifade Mountant with DAPI (Thermo Fisher) or Vectashield with DAPI (Vector Labs), imaged on a Zeiss fluorescence microscope using AxioVision or Zen lite (Blue edition) software and analyzed using Fiji (ImageJ) for mean fluorescence intensity.

Bacterial translocation measurement

Spleens, mesenteric and joint-draining (axillary) lymph nodes were isolated in a sterile fashion from early-disease and arthritic K/BxN mice. Bacterial 16 s rDNA was extracted from the digested tissues using a QIAmp DNA mini kit (51304, QIAGEN) according to the manufacturer's instructions. Briefly, samples were digested overnight in lysis buffer and Proteinase K and then homogenized using Tissue Lyser (QIAGEN). The resulting supernatant containing 16 s bacterial rDNA was purified and frozen at −20°C. The samples were quantified for the V3/V4 16 s rDNA regions by qPCR (F primer: 5′–TCCTACGGGAGGCAGCAGT, R primer: 5′-GGACTACCAGGGTATCT AATCCTGTT) using the QuantiTect SYBR Green PCR kit as per manufacturer's instructions (QIAGEN).

Arthritic cell transfer and bacterial reconstitution

Naive NOD *scid* gamma (NSG) mice were either treated with antibiotics: ampicillin (1 g/L, A9393, Sigma-Aldrich), neomycin (1 g/L, N1876, Sigma-Aldrich), metronidazole (1 g/L, M3761, Sigma-Aldrich), and vancomycin (0.5 g/L, V2002, Sigma-Aldrich); or kept on autoclaved water for three weeks. Splenocytes were isolated from arthritic K/BxN mice or NOD controls, pooled, and transferred i.v. (60 million cells/mouse) into control, antibiotics-treated mice or NSG mice reconstituted with gut microbiota from K/BxN or KRN mice as described below.

For bacterial reconstitution, NSG mice were treated with antibiotics for two weeks and changed onto autoclaved water for 24 h before bacterial reconstitution. Feces from arthritic K/BxN or control (naive KRN) mice were collected fresh, homogenized, diluted in PBS and gavaged to NSG mice (0.06 mg feces/mouse) 7 days prior and on the day of cell transfer.

Intestinal epithelial cell isolation

For the isolation of intestinal epithelial cells (IEC), PP and fat were removed from the small intestines which were separated into duodenum, jejunum and ileum. Intestinal tissues from SI and colons were cleaned in HBSS, cut longitudinally and into 2-5 mm pieces, and incubated in HBSS supplemented with 5% FCS and 2.5 mM EDTA for 40 min at 180 rpm and 37°C. The IECs were passed through a 100 μm cell strainer

and washed x2 in cRPMI. The debris and remaining fat from the intestinal sections were removed using a 25%–40% isotonic Percoll gradient spin (10 min, 600 g, 21°C) and the interface contained the epithelial cells. IECs were then washed in cRPMI and counted, and further used for FACS staining or mRNA extraction for qPCR.

Gene expression by RT-PCR

RNA was extracted from IECs according to the manufacturer's instructions (Arcturus Picopure RNA isolation kit, Applied Biosystems) and cDNA synthesis from a maximum of 500 ng of mRNA/sample was performed using iScript (Biorad). qPCR reactions for *il10ra* (QuantiTect, QIAGEN) and the housekeeping gene *hprt* (F primer: 5′-TTTGCTG ACCTGCTGGATTAC-3′, R primer: 5′-CTTTTATGTCCCCCGTTGACT-3′) were performed using iQ SYBR® Green. Samples were assayed in duplicate on an Opticon RT-qPCR machine (MJ Research) and the relative expression was calculated using the ΔC(t) method.

Small intestine organoid culture

Organoid establishment and maintenance were done similarly to published protocols[68,69]. Briefly, the proximal section of the small intestine was cut longitudinally, washed in ice-cold PBS without Mg^{2+} and Ca^{2+} (GIBCO), and the villi were scraped off, followed by further washing in PBS. The intestine was cut in 2-4 mm pieces and transferred into 50 mL Falcon tubes to be washed 2-3 times in PBS by pipetting up and down until the supernatant is clear. The clean intestinal sections are moved into new tubes with 2mM EDTA in PBS and incubated at 4°C for 30 min on a roller shaker for crypt isolation. Isolated crypts are then thoroughly washed and counted for plating in Matrigel (Corning) at approximately 300-500 crypts/30 μL Matrigel/well. Upon the solidification of the Matrigel droplets (10 min at 37°C), culture medium was added to each well: Advanced DMEM/F12 (GIBCO) containing 1 × penicillin/streptomycin (Sigma), 2 mM GlutaMAX, 10 mM HEPES, 1 × N-2 supplement, 1 × B-27 supplement (all Thermo Fisher), 1 mM N-acetylcysteine (Sigma), supplemented with epithelial growth factor (50 ng/ml, Peprotech), 1 μg/ml of human recombinant R-spondin (Biolegend), 0.1 μg/ml murine recombinant Noggin (Peprotech).

Recombinant IFNγ and IL-10 (Biolegend) were added to organoids overnight at 1 ng/ml to investigate their effect on IL-10R expression on organoid epithelial cells, stained and analyzed by flow cytometry as above.

Generation of bone marrow chimeras

Chimeric mice and the appropriate controls were generated. Briefly, recipient WT or IL-10R$^{-/-}$ mice received 9 Gy of γ-irradiation via a cesium source and were allowed to equilibrate for 5 h before being administered i.v. with 10^7 donor bone marrow cells. To restrict deficiency of IL-10R only on epithelial (non-hematopoietic) cells, the irradiated IL1-0R$^{-/-}$ mice received WT bone marrow (hematopoietic cells) and all the chimeric mice were left to reconstitute for at least 6 weeks prior to being used for AIA induction.

Modulation of intestinal permeability using AT-1001

AT-1001, a synthetic zonulin antagonist, was kindly provided by A. Fasano (Massachusetts General Hospital). Mice received 2 daily oral gavages of 50 μg of AT-1001 diluted in 150 μL water, or 150 μL water as negative control, for 8 days from the subcutaneous mBSA/CFA injection for AIA induction (described in Figure S6A).

Modulation of immune cell recirculation to the intestine

Vercirnon (Chemocentryx), a commercially available drug against chemokine receptor CCR9, was formulated as a suspension in 10% Kolliphor® EL (Sigma). 200 μg in 250 μL per mouse (10 mg/kg) were injected twice daily intra-peritoneally throughout AIA induction over the same timescale as AT-1001 treatment in Figure S6A.

16S rDNA sequencing

Bacterial sequencing was performed as previously described[70]. Briefly, 20-50 mg of fecal material was extracted using the QIAmp DNA mini kit (QIAGEN) per manufacturer's protocol with an additional cell disruption step by bead beating using lysing matrix E (MP Biomedicals) at 50 Hz for 1 minute (Tissuelyser LT, QIAGEN). Barcoded primers spanning the V3-V4 region of the 16S rRNA gene were designed as described previously[66] to include an Illumina adaptor, an 8-nucleotide barcode sequence, a 10-nucleotide pad sequence, a 2 nucleotide linker, and a gene-specific primer: 341F-CCTACGGGNGGCWGCAG or 805R- GACTACHVGGGTATCTAA TCC. (Sigma-Aldrich, Dorset, UK). Extracted DNA samples were amplified with different barcode combinations using the Taq Core PCR kit (QIAGEN) as per manufacturer's instructions. Samples were then pooled to create libraries with approximately equal concentrations of 16S rRNA amplicons from each sample, which were quality checked, sequenced, and analyzed as previously described[70,66].

QUANTIFICATION AND STATISTICAL ANALYSIS

All data are expressed as mean $\pm$ SEM. Clinical and histological scoring were performed blinded. For *in vivo* studies in the AIA model, power calculations were performed on data showing mean maximum WT arthritic knee swelling of 2 mm with an SD of 0.39 mm, and an expected test group arthritic knee swelling of $\pm$ 0.6 mm. Group sizes of three mice or above were sufficient to reach a statistical power of at least 80% (http://www.statisticalsolutions.net/pss_calc.php). To assess statistical significance, one-way ANOVA (comparison of $\geq$ 3 groups), two-way ANOVA and paired or unpaired Student's t test (comparison between 2 groups) were carried out. Bonferonni corrections were applied for multiple comparisons using ANOVA, unless otherwise stated. All data had a normal distribution, as determined by D'Agostino-Pearson omnibus normality testing. For all figures, *p* values are represented as followed: $*p < 0.05$; $**p < 0.01$; $***p < 0.001$, $****p < 0.0001$. All data, barring the immunofluorescence and histology scans, were analyzed using GraphPad Prism 7 (La Jolla, CA, USA).

Med, Volume *2*

Supplemental information

Intestinal barrier dysfunction plays an integral role in arthritis pathology and can be targeted to ameliorate disease

Diana E. Matei, Madhvi Menon, Dagmar G. Alber, Andrew M. Smith, Bahman Nedjat-Shokouhi, Alessio Fasano, Laura Magill, Amanda Duhlin, Samuel Bitoun, Aude Gleizes, Salima Hacein-Bey-Abina, Jessica J. Manson, Elizabeth C. Rosser, The ABIRISK Consortium, Nigel Klein, Paul A. Blair, and Claudia Mauri

Supplemental Table 1. Summary data of patients and healthy controls. Related to Figure 1.

**Index**	**RA (n=167)**	**Early RA (n=8)**	**Pre-RA (n=9)**	**HC (n=34)**
Female (%)	77.77%	100%	100%	70.83%
Age	52.97±15.88	44.29±8.95	51.143±14.4	49.94±17.6
Smoking	21.66% (n=60)	Unk	Unk	27.27% (n=22)
Infections	3 (2.38%)	0	0	N/A
Metabolic disorders	11 (8.73%)	3 (42.86%)	2 (28.57%)	N/A
ESR (mm/h)	17.22±20.42	25.5±7.5 (6 Unk)	N/A	N/A
CRP (g/L)	7.44±15.91	12.54±19.12	N/A	N/A
DAS28 score	3.46±1.62	2.725±0.275 (6 Unk)	N/A	N/A
RF+	80.36% (n=56)	87.5%	88.89%	N/A
CCP+	73.21% (n=56)	100%	44.45%	N/A
**Treatments**				
NSAIDs	18 (10.78%)	1 (12.5%)	1 (11.11%)	N/A
DMARDs	101 (60.47%)	2 (25%)	2 (22.22%)	N/A
Glucocorticoids	19 (11.38%)	1 (12.5%)	0	N/A
Biologics - Anti-TNFα (Adalimumab, Infliximab, Etanercept)	46 (27.55%)	0	0	N/A
Biologics - Anti-IL-6R (Tociluzimab)	7 (4.19%)	0	0	N/A
Biologics - Anti-CD20 (Rituximab)	7 (4.19%)	0	0	N/A
Untreated	18 (10.78%)	4 (50%)	6 (66.67%)	N/A

Unk- Unknown, ESR- erythrocyte sedimentation rate, CRP- C-reactive protein, DAS28- 28 Joint disease activity score, RF- rheumatoid factor, CCP - cyclic citrullinated peptide, NSAID- non-steroidal anti-inflammatory drug, DMARD- disease modifying anti-rheumatic drugs.

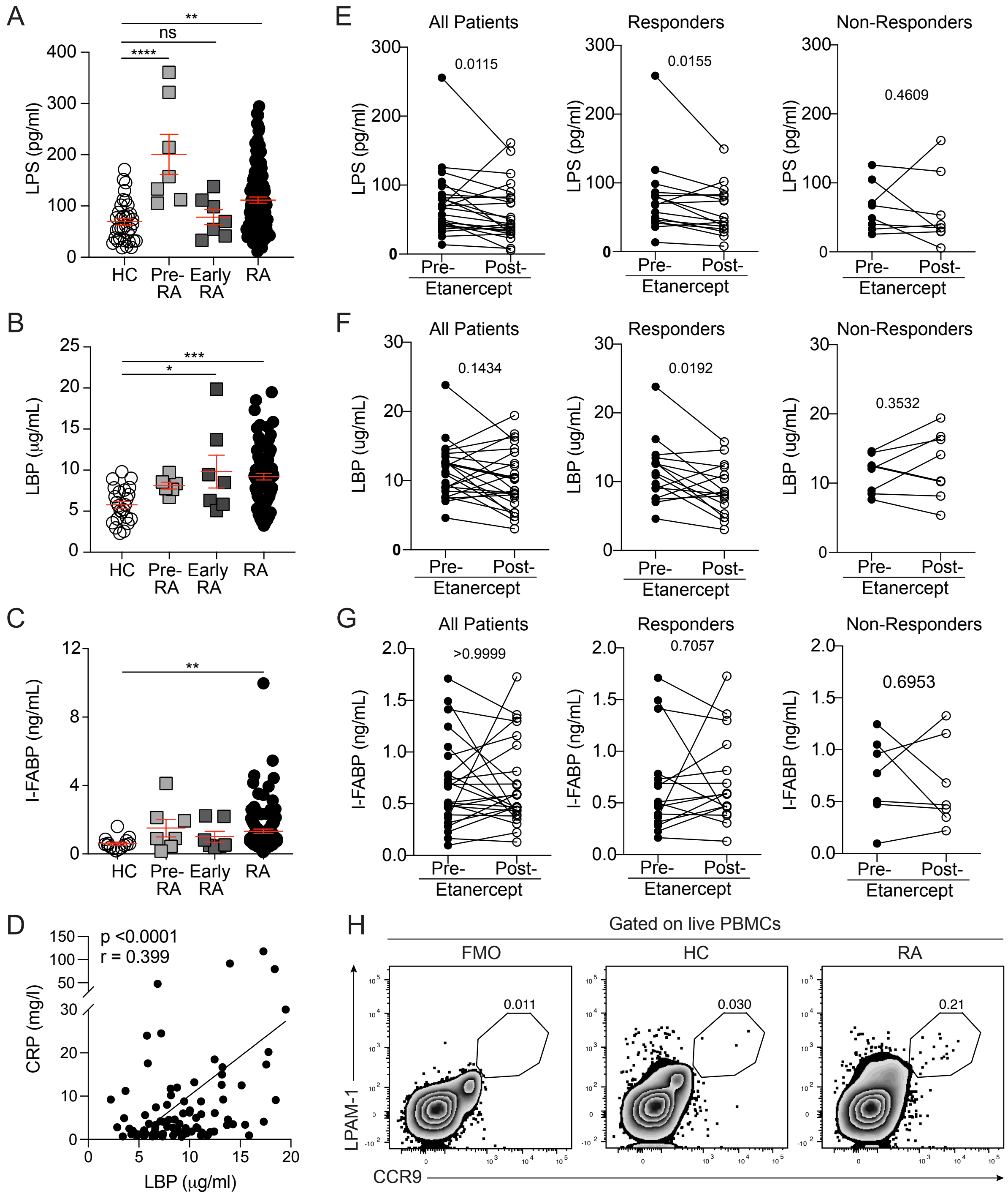

Supplemental Figure 1

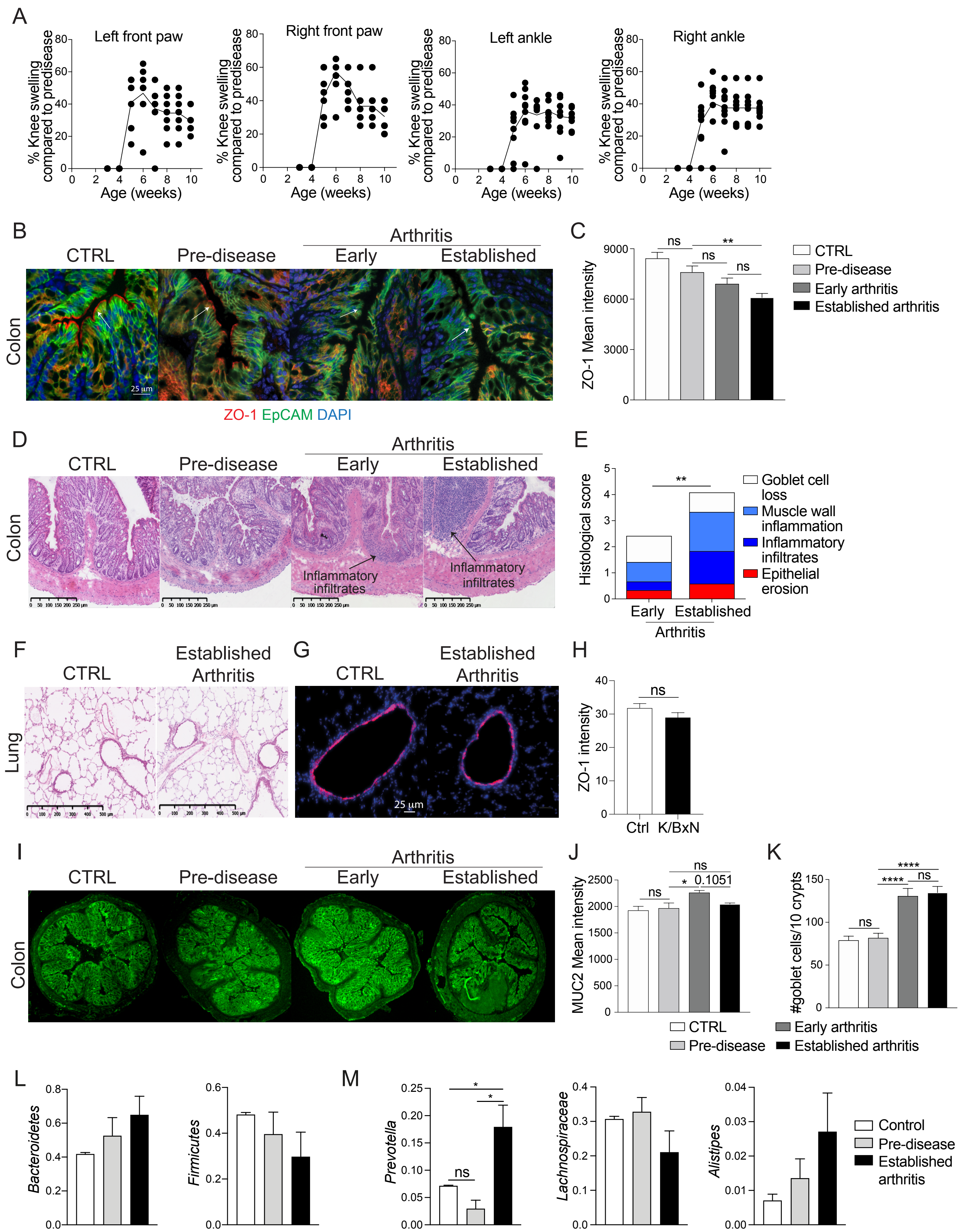

Supplemental Figure 2

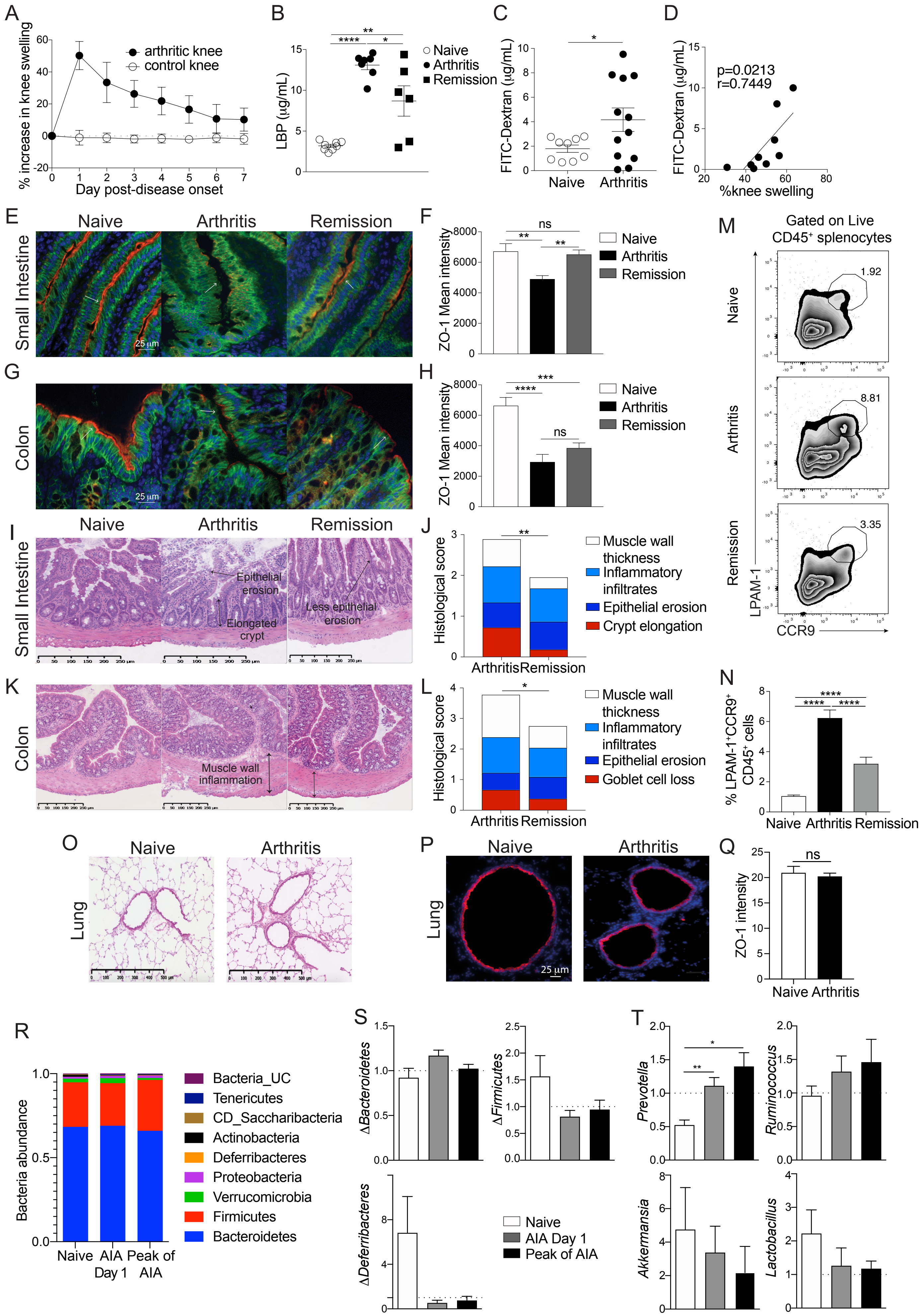

Supplemental Figure 3

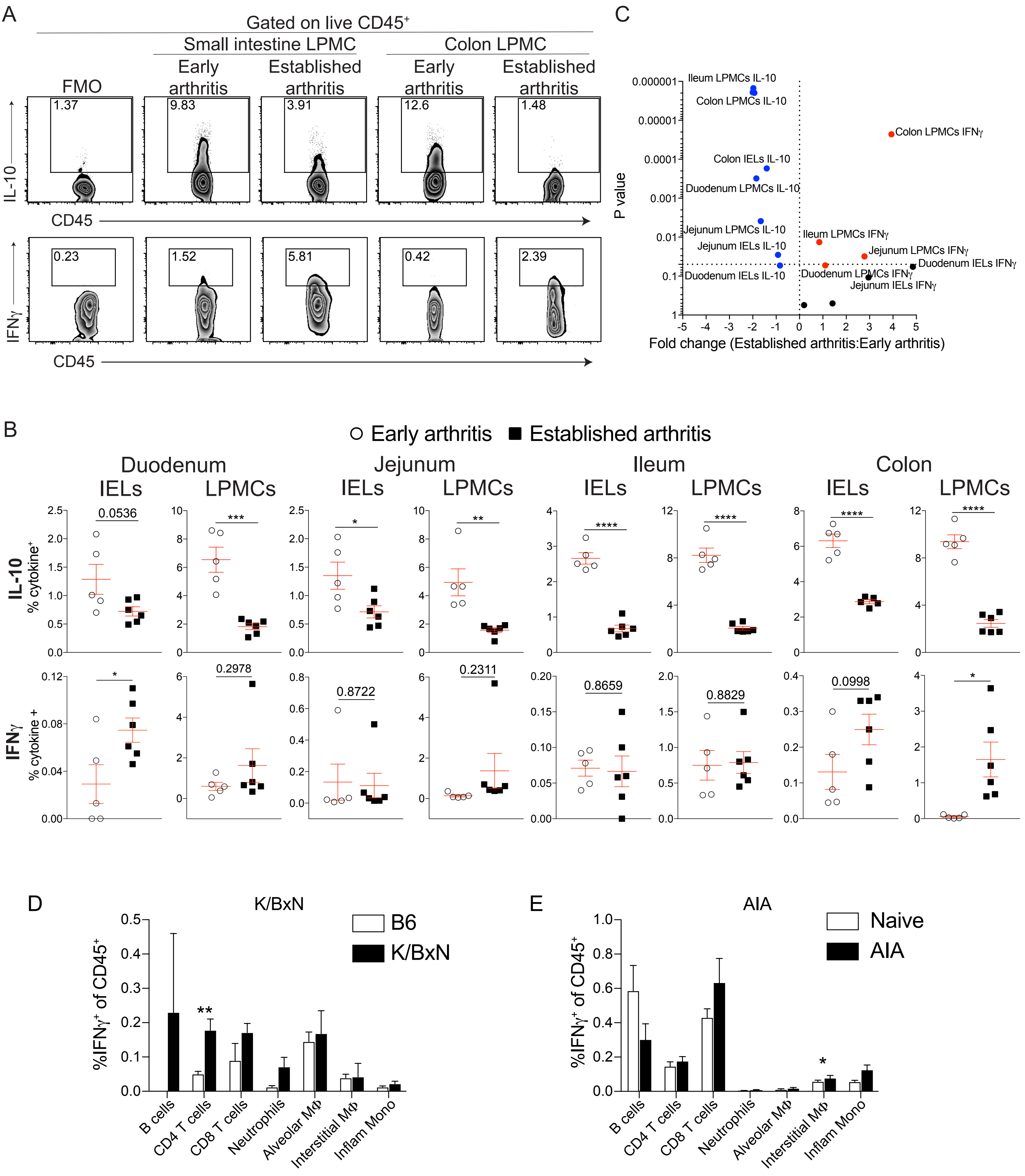

Supplemental Figure 4

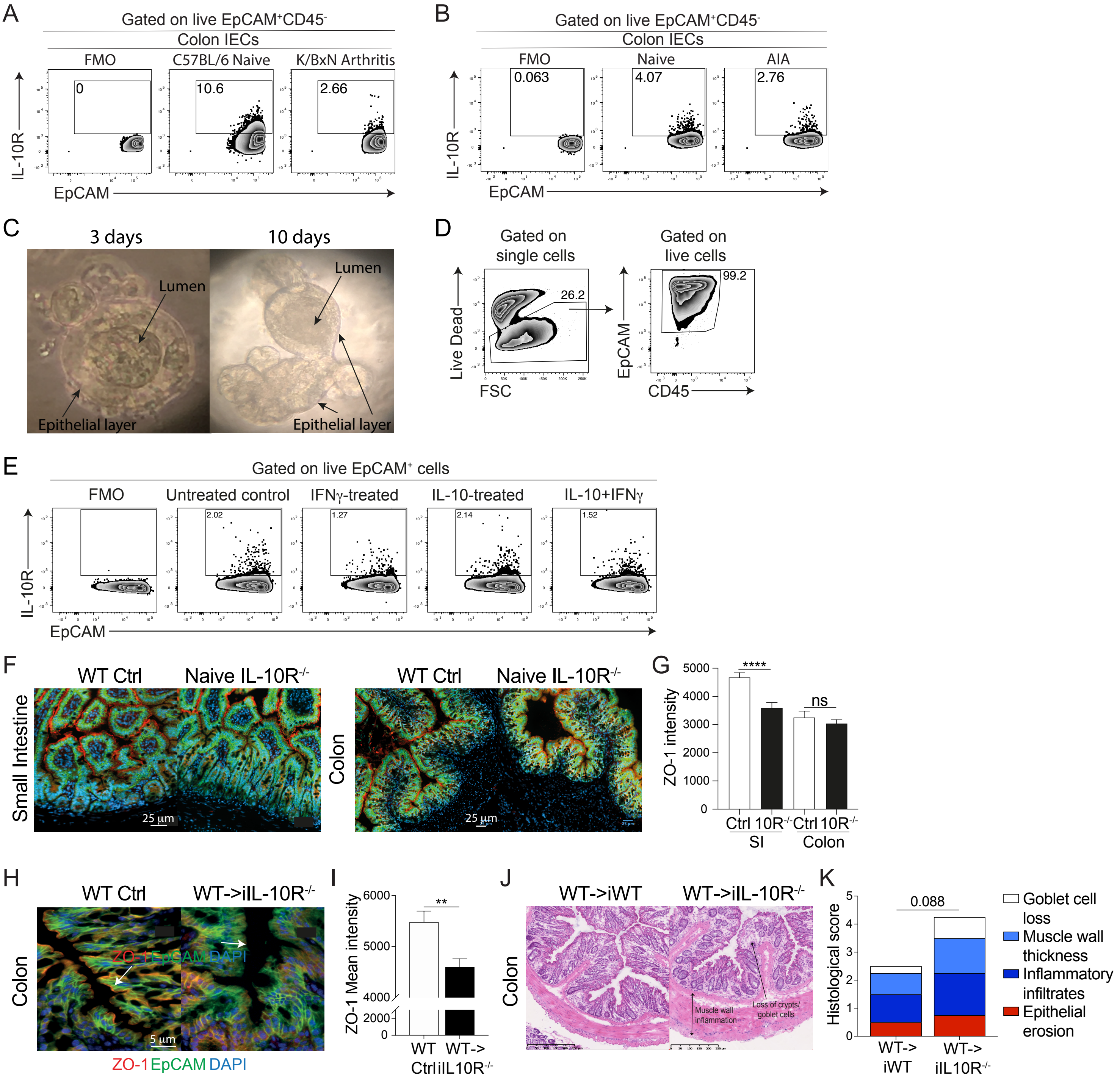

Supplemental Figure 5

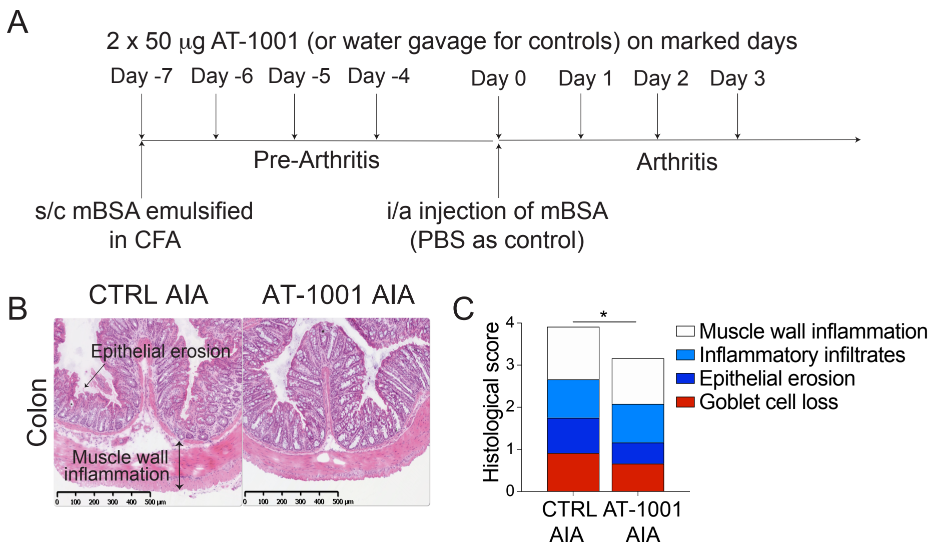

Supplemental Figure 6

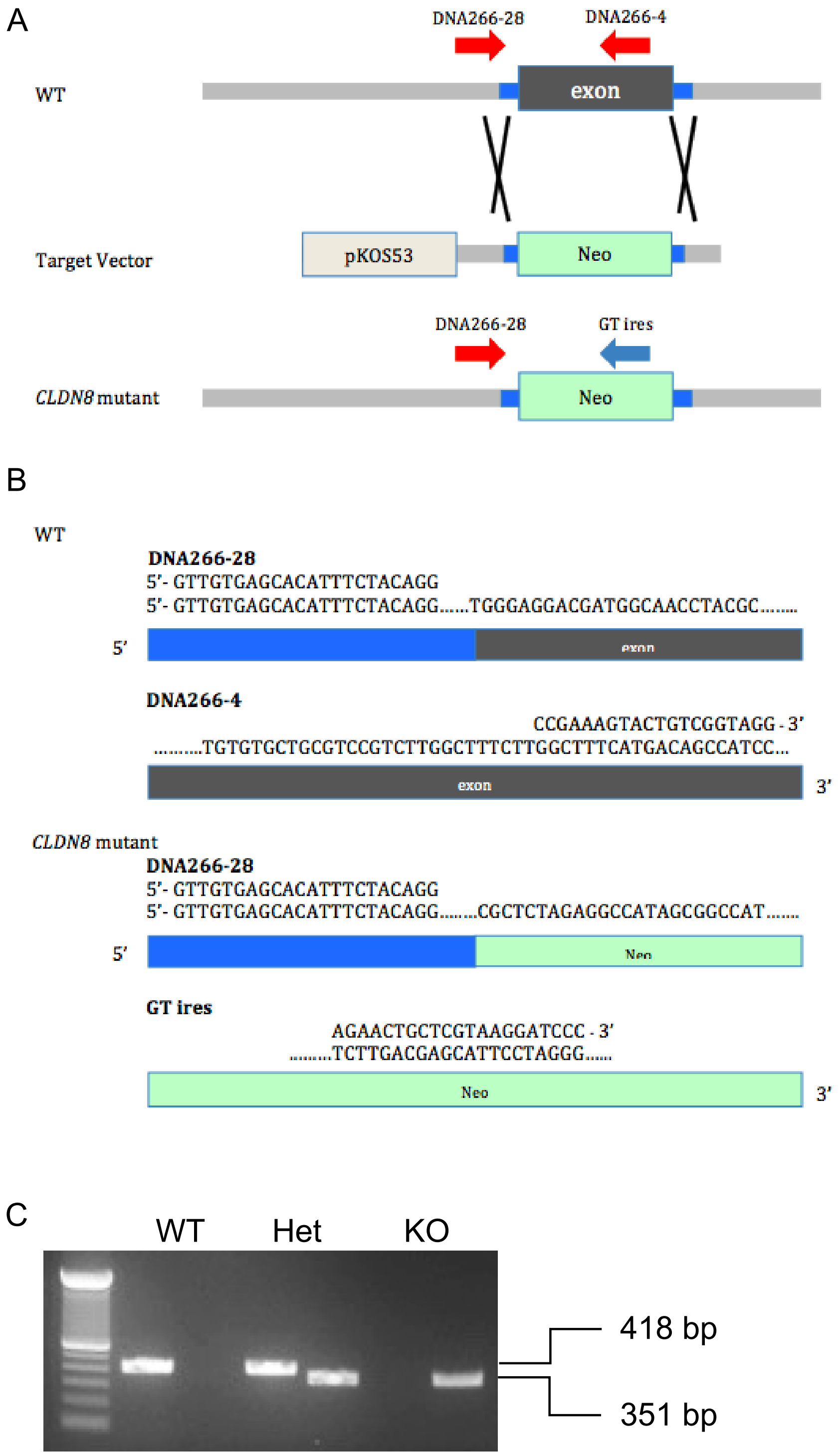

Supplemental Methods Figure 1

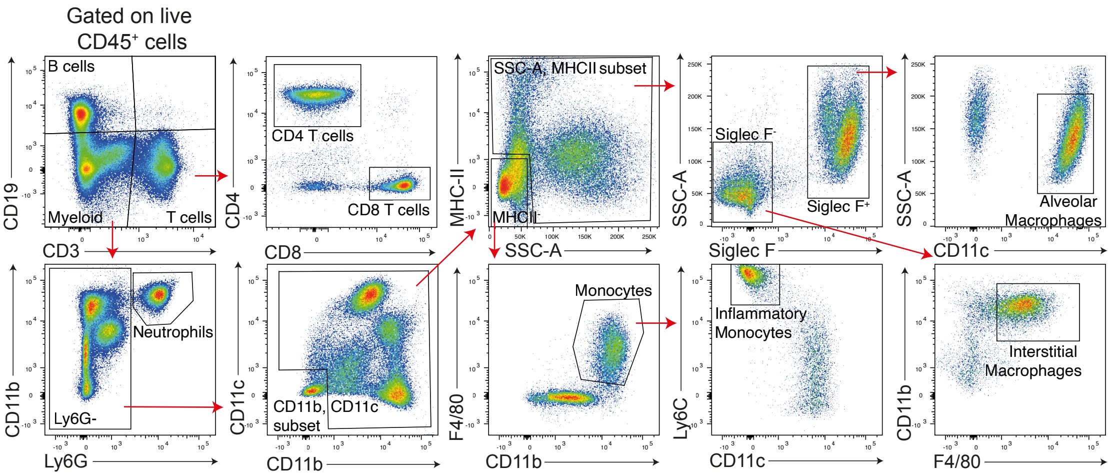

Supplemental Methods Figure 2

Supplemental Figure 1. NSAIDs do not have a significant effect on intestinal permeability, whereas etanercept partly corrects it. Related to Figure 1. (A-C) Concentration of **(A)** LPS, **(B)** LPS-binding protein (LBP) and **(C)** I-FABP in the serum of healthy individuals (HC, n=34) and in patients with pre-rheumatoid arthritis (pre-RA; n=7), early RA (n= 7) and RA patients excluding those treated with NSAIDs (n=99), as measured by ELISA. **(D)** Correlation between LBP and CRP levels in the serum of RA patients (n=124). **(E-G)** Concentration of (E)LPS, **(F)** LBP, and **(G)** I-FABP in the serum of RA patients pre- and post-etanercept treatment (approximately 6 months post-treatment). Left – all patients (n=24), Centre – treatment responders (ΔDAS28>1.2, n=16), Right – non-responders (ΔDAS28<1.2, n=8). **(H)** Representative FACS plots displaying LPAM-1^{+}CCR9^{+} live peripheral blood mononuclear cells (PBMC) isolated from RA patients and healthy controls (HC). *P < 0.05, **P < 0.01, ***P < 0.001. (**A, B, C**) one-way ANOVA with multiple comparisons calculated by Tukey's test. (**D**) Pearson's correlation. (**E, F, G**) paired t test. Data represent mean ± S.E.M.

Supplemental Figure 2. Mice with arthritis display colonic inflammation and gut microbial dysbiosis. Related to Figure 2 and Figure 3. (A) Representative time course of knee swelling in K/BxN mice with spontaneous arthritis. Knee swelling displayed as a percentage increase of knee size as compared to pre-disease initiation (n=5/group). **(B-E) (B)** Representative ZO-1 staining, **(C)** mean ZO-1intensity, **(D)** representative H&E staining and **(E)** histological scores for the colon of K/BxN mice throughout arthritis development and compared to naïve C57BL/6 control. **(F-H) (F)** Representative H&E staining, **(G)** representativeZO-1 staining, and **(H)** mean ZO-1 intensity for lungs from K/BxN mice with established arthritis and CTRL naïve mice. **(I-K) (I)** Representative MUC2 staining, **(J)** mean MUC2 intensity and **(K)** goblet cell counts from PAS staining for the colon of K/BxN mice throughout arthritis development and compared to naïve C57BL/6 control. **(L)** Bar charts showing the abundance of the noted bacterial phyla in samples collected from K/BxN mice at pre-disease (3 wk old, n=4) and established arthritis (6 wk old, n=4) stages, compared to Control (KRN, n=3). **(M)** Bar charts showing the abundance of the noted bacterial genera in samples collected from K/BxN mice at pre-disease (3 wk old, n=4) and established arthritis (6 wk old, n=4) stages, compared to Control (KRN, n=3). *P < 0.05, **P< 0.01, ****P < 0.0001, ns: no significance. (**C, J, K, L, M**) one-way ANOVA, (**E**) two-way ANOVA, (**H**) unpaired t test. For panels **A-K** one of two experiments is shown. Data represent mean ± S.E.M.

Supplemental Figure 3. Mice with AIA display increased gut permeability and inflammation and gut microbial dysbiosis from day 1 of arthritis. Related to Figure 2 and Figure 3. (A) Representative time course of knee swelling in AIA in C57BL/6 WT mice. Knee swelling displayed as percentage increase of knee size as compared to pre-disease induction (n=5/group). **(B)** Concentration of LBP in the serum of WT naïve, arthritic mice (AIA day 3), and mice in remission from arthritis (AIA day 7) (n=5/group). **(C)** Levels of FITC-dextran in the serum of WT naïve (n=9) and arthritic (n=12) mice (AIA day 3). **(D)** Correlation between FITC-dextran in the serum and %knee swelling in mice at day 3 of AIA (n=9). **(E-L) (E, G)** Representative ZO-1 staining, **(F, H)** mean ZO-1 intensity, **(I, K)** representative H&E staining and **(J, L)** histological scores for SI and colon, respectively, from WT naïve and arthritic mice

(AIA day 3) and mice in remission from arthritis (AIA day 7). **(M, N) (M)** Representative FACS plots and **(N)** bar chart showing the mean expression of LPAM-1 and CCR9 by total $CD45^+$ splenocytes from naïve WT mice, arthritic mice, and mice in remission from arthritis (n=3/group). **(O-Q) (O)** Representative H&E staining, **(P)** representative ZO-1 staining, and **(Q)** mean ZO-1 intensity for lungs from WT naive and arthritic mice (AIA day 3). **(R)** Bar chart showing relative bacterial composition at phylum level in samples collected from mice at AIA day 1 and Peak of AIA (day 3), compared to naive mice. **(S)** Bar charts showing the change in the abundance of the noted bacterial phyla between samples collected at AIA day 1 from naive and arthritic mice, and at Peak of AIA from arthritic mice, compared to their abundance in the same mice at days 1-4 (prior to arthritis induction). **(T)** Bar charts showing the abundance of the noted bacterial genera in samples collected at AIA day 1 and Peak of AIA (day 3), compared to naive mice. (n=10/group). *P < 0.05, **P < 0.01, ***P < 0.001, ****P < 0.0001, ns: no significance. (**B, F, H, N, S, T**) one-way ANOVA, (**C, Q**) unpaired t test, (**D**) Pearson's correlations, (**J, L**) two-way ANOVA. UC - Unclassified, CD - Candidatus. For panels **A-Q** one of two experiments is shown. Data represent mean ± S.E.M.

Supplemental Figure 4. Increased IFNγ and decreased IL-10 expression in the guts of arthritic K/BxN mice. Related to Figure 4. (A) Representative FACS plots displaying cytokine production by total $CD45^+$ lamina propria mononuclear cells (LPMC) isolated from the small intestine or colon of early-disease K/BxN mice and K/BxN mice with established arthritis. **(B)** Graphs displaying cytokine expression and **(C)** volcano plot showing the fold change in cytokine expression by total $CD45^+$ intra-epithelial lymphocytes (IEL) and lamina propria mononuclear cells (LPMC) isolated from the noted tissues of early-disease K/BxN mice (n=5) and mice with established arthritis (n=6), as assessed by flow cytometry. **(D, E)** Bar charts showing IFNγ expression by the noted immune cell subsets isolated from lungs of **(D)** K/BxN and C57BL/6 control mice, and from **(E)** C57BL/6 arthritic and naive mice (n=5/group), as assessed by flow cytometry. MΦ- Macrophage; Inflam Mono- Inflammatory Monocytes.*P < 0.05, **P < 0.01, ***P < 0.001, ****P < 0.0001 (**B**, **D**, **E**, unpaired t test). Data represent mean ± S.E.M.

Supplemental Figure 5. IL-10R expression by IECs ex vivo and organoids in vitro, ZO-1 expression on global IL-10R-/- and gut inflammation in non-hematopoietic cell IL-10R-/- chimeric mice. Related to Figure 4. (A) Representative FACS plots of IL-10R on colonic intestinal epithelial cells in naïve C57BL/6 control versus K/BxN arthritic mice and in **(B)** naïve versus arthritic (day 3 of AIA) C57BL/6 mice. **(C)** Representative brightfield scans of growing organoids: left - budding organoids after 3 days in culture, right - mature organoids after approximately 10 days in culture. **(D)** Representative FACS plots showing organoid survival (left) and epithelial cell purity (right). **(E)** Representative FACS plots showing IL-10R expression by epithelial cells isolated from organoids treated with recombinant IFNγ and/or IL-10, or left untreated. **(F, G)** Representative ZO-1 staining and mean ZO-1 intensity from SI and colon of naïve IL-10R-/- mice and WT control. **(H, I)** Representative ZO-1 staining and mean ZO-1 intensity from colon of naïve non-hematopoietic cell IL-$10R^{-/-}$ chimeric mice (WT->iIL-$10R^{-/-}$) and WT control. **(J, K)** Representative H&E staining and histological scores of the colon of WT->iIL-$10R^{-/-}$ and WT->iWT chimeric mice on day 3 of arthritis. **P < 0.01, ****P < 0.0001. (**G, I**) unpaired t test, (**K**) two-way ANOVA. For all panels one of two experiments is shown. Data represent mean ± S.E.M.

Supplemental Figure 6. Modulating gut permeability using AT-1001 improves gut pathology. Related to Figure 6. (A) Schematic of AT-1001 treatment regime for mice during induction of arthritis (s/c- subcutaneous, i/a- intra-articular). **(B)** Representative H&E staining and **(C)** mean histological scores of the colon of untreated and AT-1001-treated arthritic mice (AIA day 3). $^*P < 0.05$. (**C**) two-way ANOVA. For all panels one of two experiments is shown. Data represent mean ± S.E.M.

Supplemental Methods Figure 1. Generation of Cldn8$^{-/-}$ mouse. Related to STAR methods Mice section. (A) Schematic of the targeting strategy for Cldn8$^{-/-}$ mice. WT Cldn8 gene has 1 exon. The target vector, pKOS-53 with a LacZ/Neo cassette, was targeted to Cldn8 gene by homologous recombination. **(B)** Primers DNA266-28, DNA266-4, and GT ires were used to distinguish WT alleles (418 bps) from mutant alleles (351 bps). **(C)** PCR verification of mouse Cldn8 gene deletion from an ear punch biopsy using primers DNA266-28, DNA266-4, and GT ires.

Supplemental Methods Figure 2. Gating strategy for immune cells isolated from arthritic and control lungs. Related to STAR methods Cell Preparation section. Representative FACS plots of subset gating for lymphocytes isolated from the lung of a naive C57BL/6 mouse.
